# Methyltransferase SMYD3 impairs hypoxia tolerance by augmenting hypoxia signaling independent of its enzymatic activity

**DOI:** 10.1016/j.jbc.2022.102633

**Published:** 2022-10-21

**Authors:** Zixuan Wang, Xiaoyun Chen, Sijia Fan, Chunchun Zhu, Hongyan Deng, Jinhua Tang, Xueyi Sun, Shuke Jia, Qian Liao, Wuhan Xiao, Xing Liu

**Affiliations:** 1 State Key Laboratory of Freshwater Ecology and Biotechnology, Institute of Hydrobiology, Chinese Academy of Sciences, Wuhan, China; 2 University of Chinese Academy of Sciences, Beijing, China; 3 College of Life Science, Wuhan University, Wuhan, China; 4 The Innovation of Seed Design, Chinese Academy of Sciences, Wuhan, China; 5 Hubei Hongshan Laboratory, Wuhan, China

**Keywords:** SMYD3, HIF1α, hypoxia signaling, ROS, zebrafish, hypoxia tolerance, DFX, deferoxamine mesylate salt, FBS, fetal bovine serum, HIF, hypoxia-inducible factor, MEF, mouse embryonic fibroblast, VHL, von Hippel-Lindau, PHD, prolyl hydroxylase, ROS, reactive oxygen species, SETSu (var) 3–9, Enhancer-of-zeste, and N-terminal Trithorax

## Abstract

Hypoxia-inducible factor (HIF)1α, a main transcriptional regulator of the cellular response to hypoxia, also plays important roles in oxygen homeostasis of aerobic organisms, which is regulated by multiple mechanisms. However, the full cellular response to hypoxia has not been elucidated. In this study, we found that expression of SMYD3, a methyltransferase, augments hypoxia signaling independent of its enzymatic activity. We demonstrated SMYD3 binds to and stabilizes HIF1α *via* co-immunoprecipitation and Western blot assays, leading to the enhancement of HIF1α transcriptional activity under hypoxia conditions. In addition, the stabilization of HIF1α by SMYD3 is independent of HIF1α hydroxylation by prolyl hydroxylases and the intactness of the von Hippel-Lindau ubiquitin ligase complex. Furthermore, we showed SMYD3 induces reactive oxygen species accumulation and promotes hypoxia-induced cell apoptosis. Consistent with these results, we found *smyd3*-null zebrafish exhibit higher hypoxia tolerance compared to their wildtype siblings. Together, these findings define a novel role of SMYD3 in affecting hypoxia signaling and demonstrate that SMYD3-mediated HIF1α stabilization augments hypoxia signaling, leading to the impairment of hypoxia tolerance.

It is well-known that oxygen profoundly affects physiology of aerobic organisms through multiple mechanisms. Molecular oxygen not only acts as the terminal electron acceptor at complex IV of the respiratory chain that yields energy during aerobic respiration and builds metabolites but also promotes to change the configuration and function of nucleic acids, sugars, lipids, proteins, and metabolites. Inadequate oxygen availability can lead to cellular dysfunction and even cell death. Under low oxygen (hypoxic) conditions, aerobic organisms utilize their cardiovascular system and respiratory system to ensure adequate oxygen delivery to cells and tissues. In addition, cells undergo adaptive changes to initiate gene expression that either enhance oxygen delivery or promote survival (1). In addition, hypoxic conditions can also trigger oxidative stress by generating uncontrolled reactive oxygen species (ROS) in mitochondria, which may pose a threat to cell survival. ROS, a generic term for a large family of oxidants derived from molecular oxygen, can be neutralized by catalase, peroxidase, and superoxide dismutase. However, under hypoxic conditions, disturbances in electron transport are associated with electron leakage from the respiratory chain, giving rise to increased ROS, which may be toxic to cells if ROS levels are not attenuated (2, 3, 4, 5).

In the process of hypoxia adaptation, the hypoxia signaling pathway mediated by hypoxia-inducible factor (HIF) plays a pivotal role (6, 7, 8, 9, 10, 11). As a key modulator of the transcriptional response to hypoxic stress, HIF is a heterodimer of bHLH-PAS proteins consisting of an O_2_-labile alpha subunit (HIFα) and a stable beta subunit (HIF1β)/(ARNT) that binds hypoxia response elements. Aerobic organisms possess three HIFα proteins, of which HIF1α and HIF2α are the most structurally similar containing two transactivation domain (N-terminal transactivation domain and C-terminal transactivation domain) (6, 10).

Under well oxygenated (normoxic) conditions, HIFα subunit is hydroxylated at two highly conserved prolyl residues by the prolyl hydroxylases (PHDs) (also called EglNs), whose activity is regulated by O_2_ availability (6, 12, 13). Hydroxylated HIFα generates a binding site for being recognized by the von Hippel-Lindau (pVHL) tumor suppressor protein complex, which is an ubiquitin ligase complex. As a result, HIFα is polyubiquitinated and subjected to proteasomal degradation. Under hypoxic conditions, PHDs activity is diminished, leading to stabilization and accumulation of HIFα proteins. Stabilized HIFα proteins dimerize with HIF1β, translocate to the nucleus, and induce transcription of genes involved in hypoxia adaptation or tolerance (6, 7). The factors affecting hypoxia signaling pathway mainly impact on HIFα protein stability (14, 15, 16). In addition, FIH (factor that inhibits HIF)-mediated asparagine hydroxylation impairs the transcriptional activity of HIF by interrupting the interaction between HIF and the transcriptional cofactor CBP/p300 (17).

In addition to oxygen-dependent hydroxylation, HIFα is also regulated by other posttranslational modifications, including ubiquitination/deubiquitination, phosphorylation, acetylation/deacetylation, SUMOylation, methylation, S-Nitrosylation, glycosylation, and neddylation (16, 18). Most of these modifications are enzymatically driven, leading to either increased or decreased HIFα stability (19, 20, 21, 22, 23, 24, 25, 26, 27, 28, 29, 30). Notably, some binding partners with enzymatic activity can also affect HIFα stability or activity independent of their enzymatic activity (31, 32).

SMYD3 is a member of the SMYD lysine methylase family containing two conserved structural domains: the catalytic Su (var) 3–9, Enhancer-of-zeste, and N-terminal Trithorax (SET) domain, which is split by a Myeloid-Nervy-DEAF1 domain (33). The SET domain of SMYD3 is comprised of two sections: the S-sequence, which may function as a cofactor binder as well as for protein–protein interactions, and the core SET domain, which functions as the primary catalytic location domain, and the C-terminal domain (33, 34, 35). *SMYD3* plays an important role in the methylation of various histone and nonhistone targets involved in tumorigenesis and affecting transcriptional regulation (36, 37, 38, 39, 40, 41, 42). In addition, it was reported previously that the oncogenic function of SMYD3 is partially independent on its methyltransferase activity (43, 44).

Whether or not SMYD3 involved in hypoxia signaling is still not understood. In this study, we show that SMYD3 interacts with HIF1α and stabilizes HIF1α independent of its methyltransferase activity, leading to the augment of the hypoxia signaling, the accumulation of ROS, and the enhancement of hypoxia-induced cell apoptosis. By zebrafish model, we found that disruption of *smyd3* facilities zebrafish hypoxia tolerance, which might be resulted from the impact of *smyd3* on hypoxia signaling.

## Results

### SMYD3 augments hypoxia signaling

**Figure 1:**
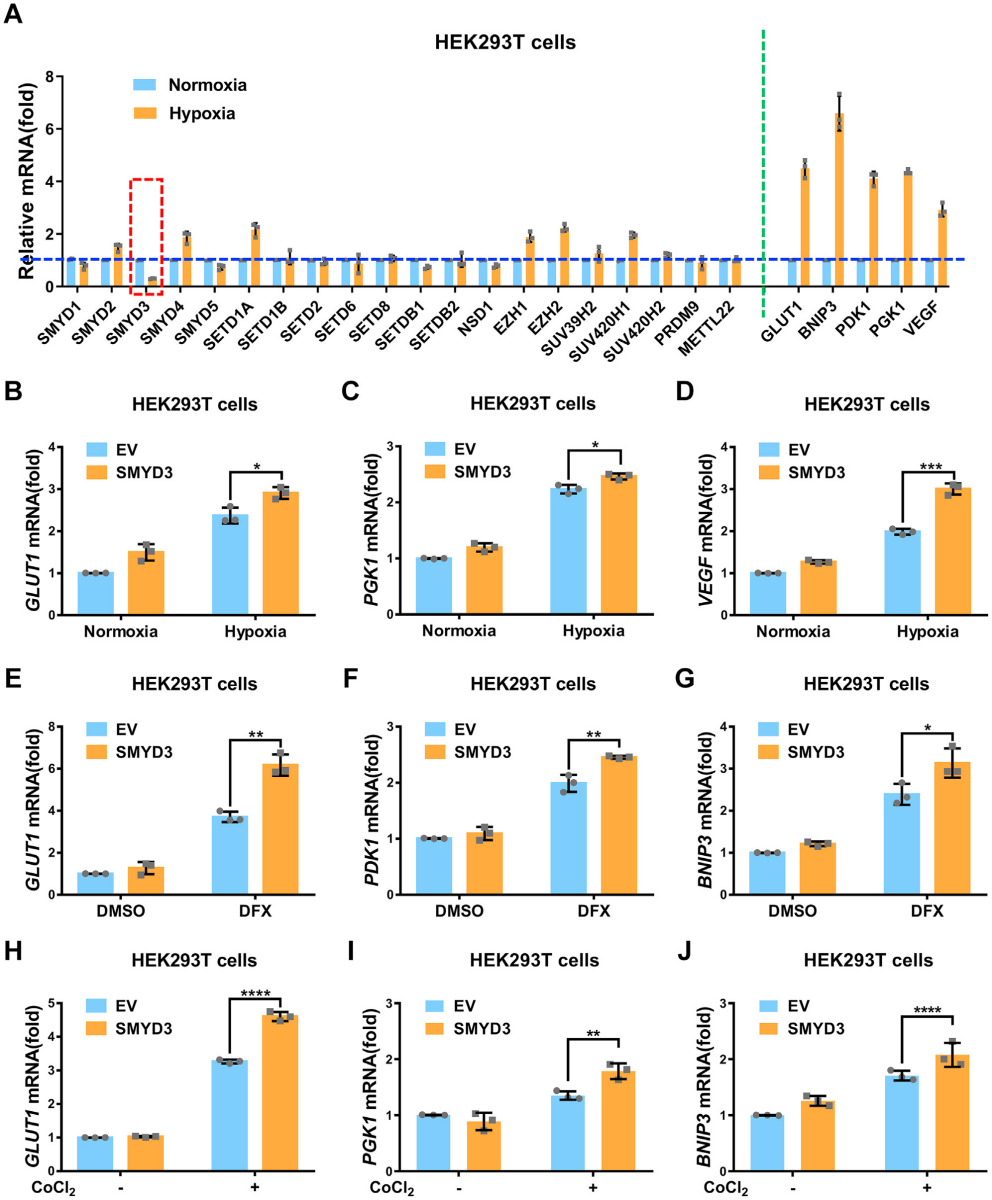
**SMYD3 augments hypoxia signaling**. *A*, quantitative real-time PCR (qPCR) analysis of mRNA levels of indicated lysine methyltransferase genes and hypoxia signaling target genes in HEK293T cells under normoxia (21% O_2_) or hypoxia (1% O_2_) for 24 h. *B*–*D*, qPCR analysis of *GLUT1* (*B*), *PGK1* (*C*), and *VEGF* (*D*) mRNA in HEK293T cells transfected with or without pCMV-SMYD3 under normoxia (21% O_2_) or hypoxia (1% O_2_) for 24 h. *E*–*G*, qPCR analysis of *GLUT1* (*E*), *PDK1* (*F*), and *BNIP3* (*G*) mRNA in HEK293T cells transfected with or without pCMV-SMYD3 and treated with DFX (150 μM) or DMSO as a control for 8 h. *H–J*, qPCR analysis of *GLUT1* (*H*), *PGK1* (*I*), and *BNIP3* (*J*) mRNA in HEK293T cells transfected with or without pCMV-SMYD3 and treated with or without CoCl_2_ (200 μM) for 8 h. EV, pCMV empty vector (control). Data show mean ± SD; Student’s two-tailed *t* test. ∗*p* < 0.05, ∗∗*p* < 0.01, ∗∗∗*p* < 0.001, ∗∗∗∗*p* < 0.0001. Data from three independent experiments.

**Supplemental Figure 1:**
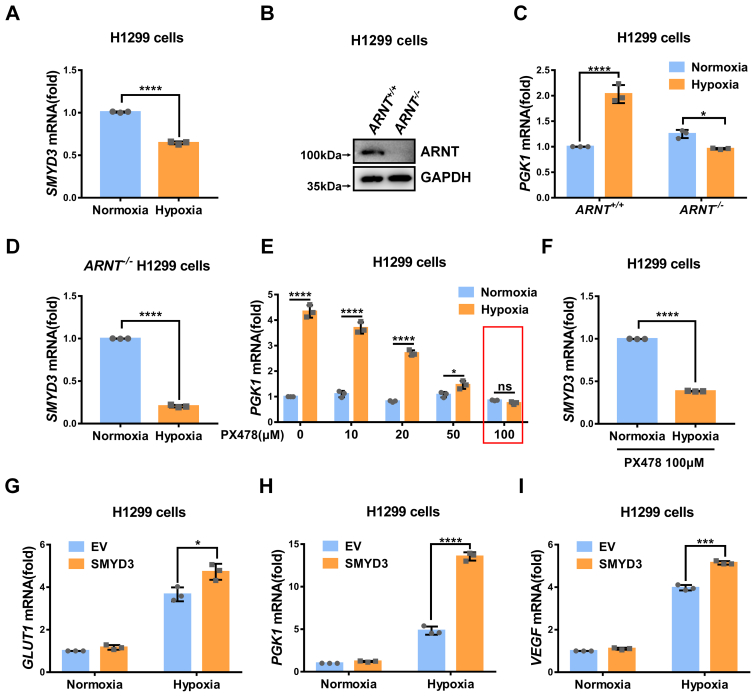


We have previously identified that the monomethyltransferase, SET7, represses hypoxia signaling by catalyzing HIF-α methylation (30). To determine whether other methyltransferases also involved in hypoxia signaling, initially, we examined expression of a series of methyltransferases in HEK293T cells under hypoxia. As shown in Fig. 1*A*, the typical hypoxia responsive genes, including *GLUT1, BNIP3, PDK, PGK1*, and *VEGF* (30, 31, 45), were greatly induced under hypoxia, suggesting the hypoxic condition was achieved expectedly. Among the methyltransferase genes tested, *SMYD2*, *SMYD4*, *SETD1A*, *EZH1*, *EZH2*, and *SUV420H1* were upregulated under hypoxia, but only *SMYD3* was significantly suppressed (Fig. 1*A*), which provoked us to further test the impact of *SMYD3* in affecting hypoxia signaling. Subsequently, we examined whether the effect of hypoxia on *SMYD3* expression is dependent of HIF signaling. In H1299 cells, the expression of *SMYD3* was significantly suppressed under hypoxia (Fig. S1*A*). However, in *ARNT*-deficient H1299 cells (*ARNT*^−/−^) (Fig. S1*B*), hypoxia failed to induce expression of *PGK1*, a typical HIF1α target gene (Fig. S1*C*) but could still suppress expression of *SMYD3* (Fig. S1*D*). In addition, we added PX478 to inhibit HIF1α activity and then checked the effect of hypoxia on *SMYD3* expression (46). When PX478 (100 μM) was added, hypoxia failed to induce expression of *PGK1* (Fig. S1*E*) but could still suppress expression of SMYD3 (Fig. S1*F*). These results suggest that the effect of hypoxia on *SMYD3* is independent of HIF signaling.

**Figure 2:**
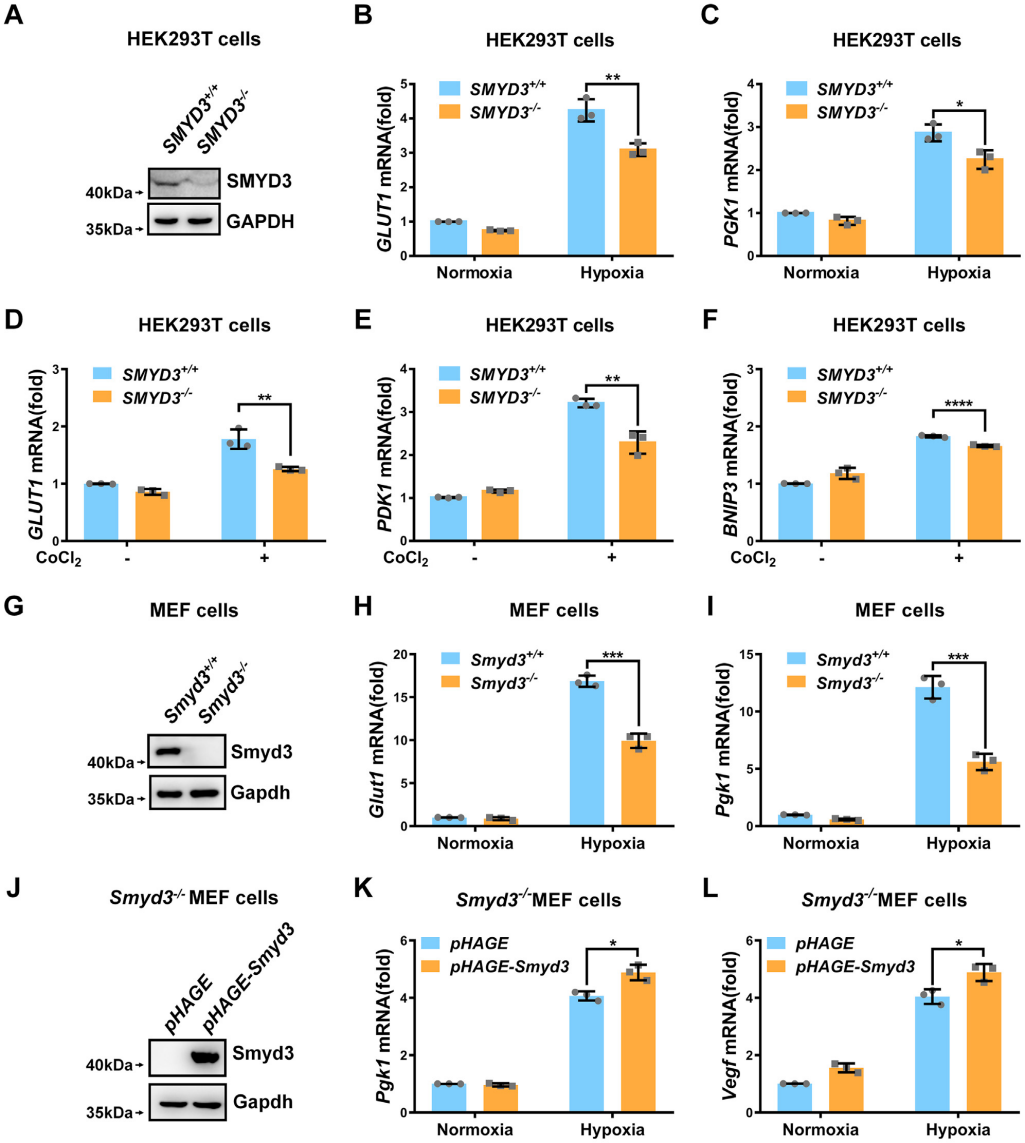
**Loss of SMYD3 diminishes hypoxia signaling**. *A*, immunoblotting of indicated proteins in *SMYD3*-deficient or wildtype HEK293T cells (*SMYD3*^−/−^ or *SMYD3*^+/+^). *B* and *C*, qPCR analysis of *GLUT1* (*B*) and *PGK1* (*C*) mRNA in *SMYD3*-deficient or wildtype HEK293T cells (*SMYD3*^−/−^ or *SMYD3*^+/+^) under normoxia (21% O_2_) or hypoxia (1% O_2_) for 24 h. Data show mean ± SD; Student’s two-tailed *t* test. ∗*p* < 0.05, ∗∗*p* < 0.01. Data from three independent experiments. *D–F*, qPCR analysis of *GLUT1* (*D*), *PDK1* (*E*), and *BNIP3* (*F*) mRNA in *SMYD3*-deficient or wildtype HEK293T cells (*SMYD3*^−/−^ or *SMYD3*^+/+^) treated with or without CoCl_2_ (200 μM) for 8 h. Data show mean ± SD; Student’s two-tailed *t* test. ∗∗*p* < 0.01, ∗∗∗∗*p* < 0.0001. Data from three independent experiments. *G*, immunoblotting of indicated proteins in *Smyd3*-deficient or wildtype MEF cells (*Smyd3*^−/−^ or *Smyd3*^+/+^). *H* and *I*, qPCR analysis of *Glut1* (*H*) and *Pgk1* (*I*) mRNA in *Smyd3*-deficient or wildtype MEF cells (*Smyd3*^−/−^ or *Smyd3*^+/+^) under normoxia (21% O_2_) or hypoxia (1% O_2_) for 24 h. Data show mean ± SD; Student’s two-tailed *t* test. ∗∗∗*p* < 0.001. Data from three independent experiments. *J*, immunoblotting of indicated proteins in *Smyd3*-deficient MEF cells reconstituted with or without wildtype *Smyd3 by* lentivirus. *K* and *L*, qPCR analysis of *Pgk1* (*K*) and *Vegf* (*L*) mRNA in *Smyd3*-deficient MEF cells reconstituted with or without wildtype *Smyd3 by* lentivirus under normoxia (21% O_2_) or hypoxia (1% O_2_) for 24 h. Data show mean ± SD; Student’s two-tailed *t* test. ∗*p* < 0.05. Data from three independent experiments. qPCR, quantitative RT–PCR; MEF, mouse embryonic fibroblast.

**Supplemental Figure 2:**
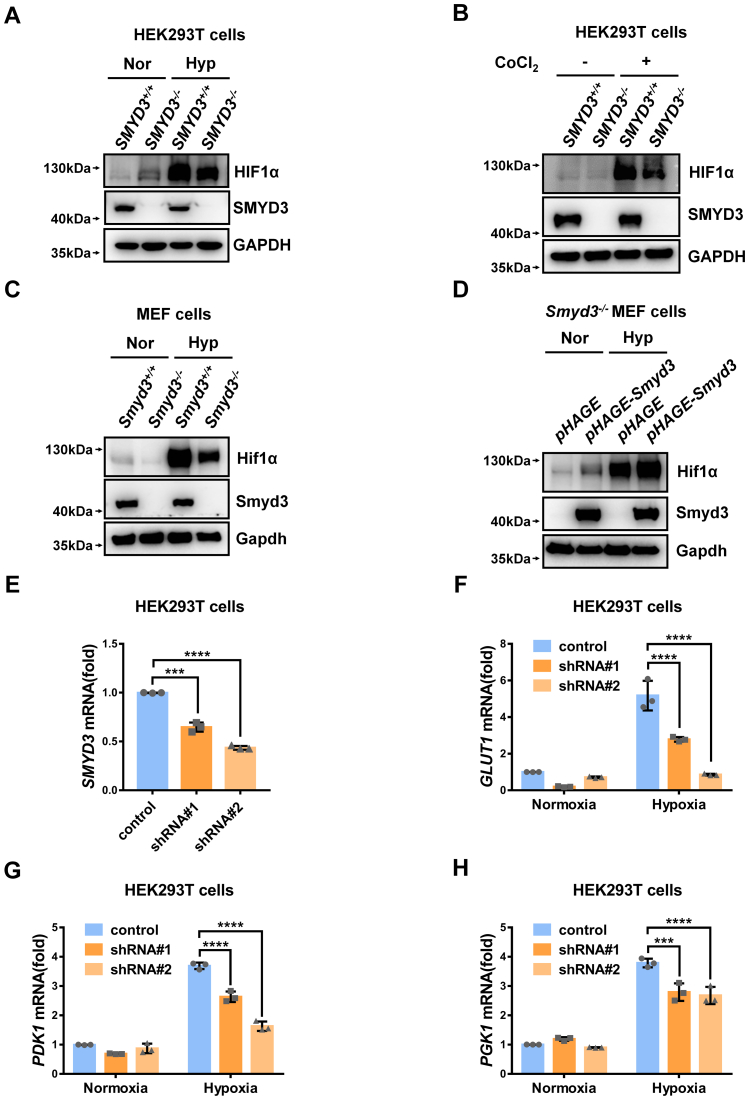


**Supplemental Figure 3:**
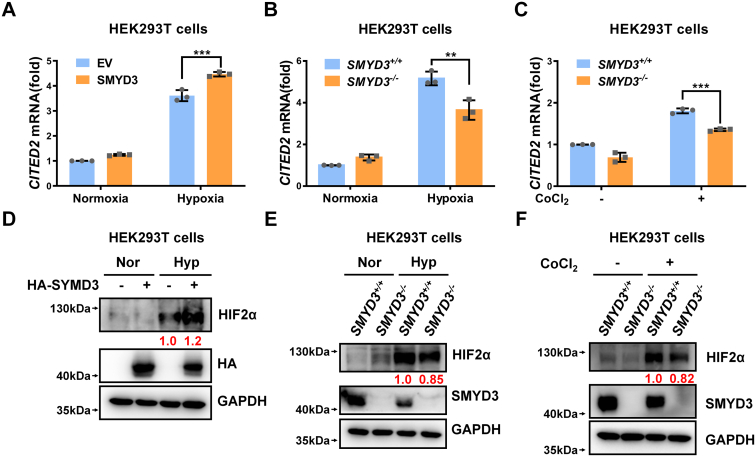


To determine the effect of *SMYD3* on hypoxia signaling, we overexpressed *SMYD3* in HEK293T cells and examined expression of hypoxia responsive genes under normoxia or hypoxia. Ectopic expression of *SMYD3* promoted expression of typical hypoxia responsive genes, including *GLUT1, PGK1*, and *VEGF*, under hypoxia (Fig. 1, *B*–*D*). To further confirm these observations, we changed direct-hypoxia treatment to the addition of deferoxamine mesylate salt (DFX) or CoCl_2_, two widely used hypoxia-mimic conditions (47, 48) and then examined the effect of *SMYD3* on hypoxia responsive gene expression. Consistently, overexpression of *SMYD3* also enhanced expression of *GLUT1*, *PDK1*, *PGK1*, and *BNIP3* (Fig. 1, *E*–*J*). SMYD3 is reported to downregulate the protein level of p53 (49), and p53 plays vital roles in hypoxia signaling (50). To exclude whether the effect of SMYD3 on hypoxia signaling was mediated by p53, we examined the effect of SMYD3 on hypoxia signaling in p53-deficient H1299 cells. Similar results were obtained by H1299 cells (Fig. S1, *G*–*I*). In contrast, knockout of *SMYD3* in HEK293T cell resulted in a reduction of expression of *GLUT1*, *PGK1*, *PDK1*, or *BNIP3* under hypoxia or CoCl_2_ treatment (Fig. 2, *A*–*F*). Moreover, expression of *Glut1* and *Pgk1* was also reduced in *Smyd3*-deficient (*Smyd3*^−/−^) mouse embryonic fibroblast (MEF) cells compared to the wildtype MEF cells (*Smyd3*^+/+^) (Fig. 2, *G*–*I*). However, reconstitution of *Smyd3* by lentivirus infection in *Smyd3*^-/-^ MEF cells recovered the induction of expression of *Pgk1* and *Vegf* compared to the empty virus control (pHAGE) (Fig. 2, *J*–*L*). HIF1α expression was confirmed by Western blot analysis (Fig. S2, *A*–*D*). In addition, knockdown of *SMYD3* by shRNAs in HEK293T cell resulted in a reduction of expression of *GLUT1, PDK1, or PGK1* under hypoxia (Fig. S2, E–*H*). Moreover, SMYD3 had similar effect on HIF2α as that on HIF1α in HEK293T cells (Fig. S3, *A*–*F*). These data suggest that *SMYD3* augments hypoxia signaling.

### SMYD3 binds to and stabilizes HIF1α, leading to an increase of nuclear HIF1α and enhancement of HIF1α-mediated target genes expression

**Figure 3:**
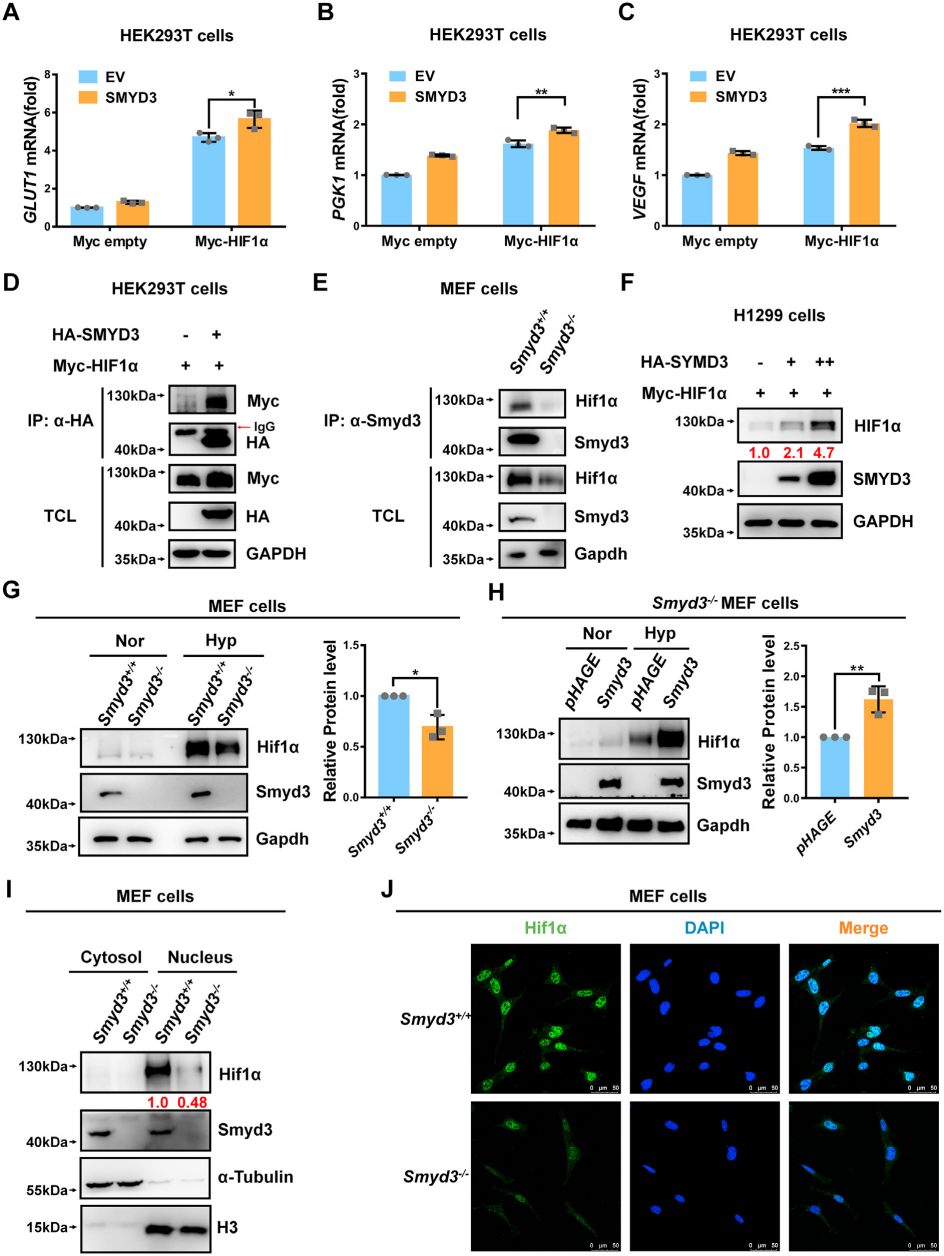
**SMYD3 binds to and stabilizes HIF1α, leading to an increase of nuclear HIF1α and enhancement of HIF1α-mediated target genes expression.***A–C*, qPCR analysis of *GLUT1* (*A*), *PGK1* (*B*), and *VEGF* (*C*) mRNA in HEK293T cells cotransfected with Myc-HIF1α or Myc empty vector (control) together with pCMV-SMYD3 or pCMV empty vector (EV) (control) for 24 h. Data show mean ± SD; Student’s two-tailed *t* test. ∗*p* < 0.05, ∗∗*p* < 0.01, ∗∗∗*p* < 0.001. Data from three independent experiments. *D*, co-immunoprecipitation of HA-SMYD3 with Myc-HIF1α. HEK293T cells were co-transfected with indicated plasmids for 24 h. Anti-HA antibody-conjugated agarose beads were used for immunoprecipitation, and the interaction was detected by immunoblotting with the indicated antibodies. *E*, endogenous interaction between Smyd3 and Hif1α. *Smyd3*-deficient or wildtype MEF cells (*Smyd3*^−/−^ or *Smyd3*^+/+^) under hypoxia for 4 h and anti-HIF1α antibody was used for immunoprecipitation. *F*, immunoblotting of exogenous Myc-HIF1α expression in H1299 cells transfected with an increasing amount of HA-SMYD3 expression plasmid (HA empty vector [-] was used as a control). *G*, immunoblotting of endogenous HIF1α expression in *Smyd3*-deficient or wildtype MEF cells (*Smyd3*^−/−^ or *Smyd3*^+/+^) under normoxia (21% O_2_) or hypoxia (1% O_2_) for 4 h. The relative intensities of Hif1α were determined by normalizing the intensities of Hif1α to the intensities of Gapdh. Data show mean ± SD; Student’s two-tailed *t* test. ∗*p* < 0.05. Data from three independent experiments. *H*, immunoblotting of endogenous Hif1α expression in *Smyd3*-deficient MEF cells reconstituted with or without wildtype *Smyd3 by* lentivirus under normoxia (21% O_2_) or hypoxia (1% O_2_) for 4 h. The relative intensities of Hif1α were determined by normalizing the intensities of Hif1α to the intensities of Gapdh. Data show mean ± SD; Student’s two-tailed *t* test. ∗∗*p* < 0.01. Data from three independent experiments. *I*, *Smyd3*-deficient or wildtype MEF cells (*Smyd3*^−/−^ or *Smyd3*^+/+^) were cultured under hypoxia for 4 h. Western blot analysis was used to detect Smyd3 and Hif1α in cytosol and nuclear fractions. *J*, confocal microscopy image of endogenous Hif1α in *Smyd3*-deficient or wildtype MEF cells (*Smyd3*^−/−^ or *Smyd3*^+/+^) under hypoxia for 4 h. Scale bar = 50 μm. MEF, mouse embryonic fibroblast; qPCR, quantitative RT–PCR; HIF, hypoxia-inducible factor.

**Supplemental Figure 4:**
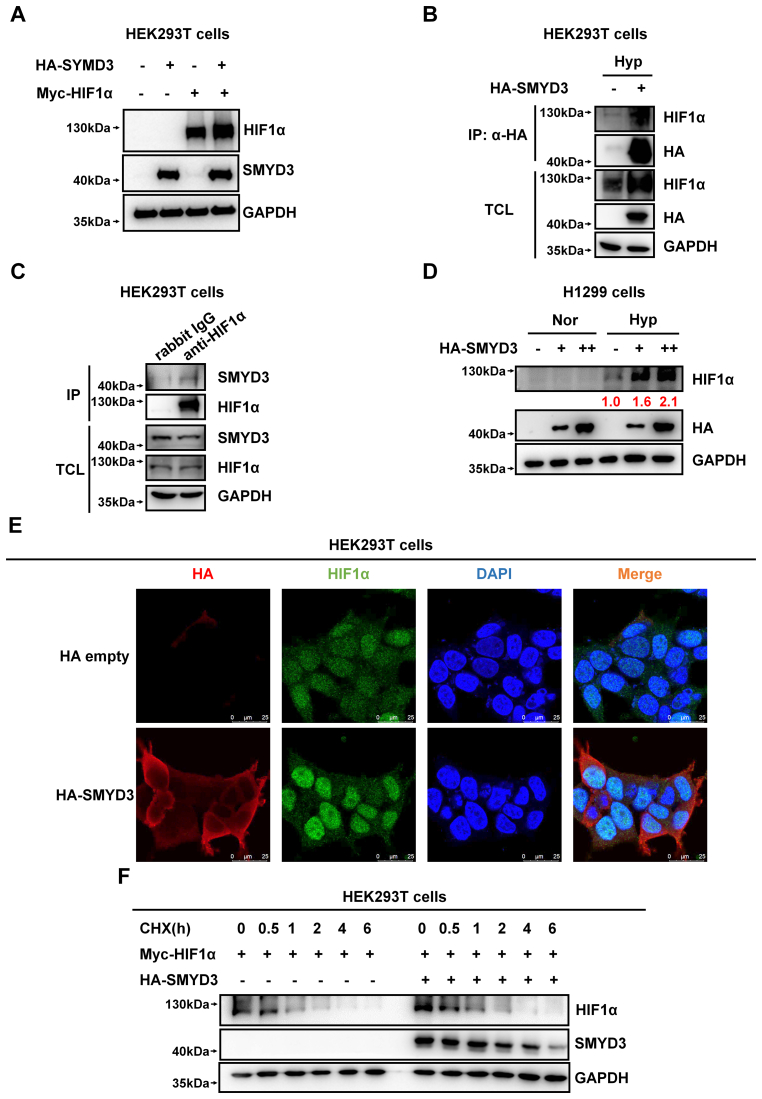


Given that HIF1α is one of the master regulators of hypoxia signaling, the enhancement of *SMYD3* on hypoxia responsive gene expression promoted us to test whether this effect is mediated by HIF1α. Co-expression of *SMYD3* together with *HIF1α* caused an induction of expression of *GLUT1*, *PGK1*, and *VEGF* mediated by ectopic expression of *HIF1α* in HEK293T cells (Fig. 3, *A*–*C*). HIF1α expression was confirmed by Western blot analysis (Fig. S4*A*).

We next examined whether SMYD3 interacted with HIF1α. Co-immunoprecipitation assays indicated that ectopic-expressed HA-SMYD3 interacted with ectopic-expressed Myc-HIF1α (Fig. 3*D*). Semiendogenous co-immunoprecipitation assays indicated that ectopic-expressed HA-SMYD3 interacted with endogenous HIF1α under hypoxia (Fig. S4*B*). Endogenous interaction between SMYD3 and HIF1α was further confirmed in HEK293T cells under hypoxia (Fig. S4*C*). In *Smyd3*^+/+^ MEF cells, but not in *Smyd3*^−/−^ MEF cells, endogenous Smyd3 interacted with endogenous HIF1α (Fig. 3*E*). Furthermore, we examined whether the protein stability of HIF1α is affected by SMYD3. Co-expression of *SMYD3* together with *HIF1α* caused HIF1α protein level was increased steadily (Fig. 3*F*). Overexpression of *SMYD3* upregulated endogenous HIF1α protein level under hypoxia (Fig. S4*D*). By contrast, the endogenous Hif1α protein level was lower in *Smyd3*-null MEF cells (*Smyd3*^−/−^) compared to that in *Smyd3*-intact MEF cells (*Smyd3*^+/+^) under hypoxia (Fig. 3*G*). Consistently, reconstitution of *Smyd3* in *Smyd3*^−/−^ MEF cells caused an increase of Hif1α protein under hypoxia (Fig. 3*H*).

Since stabilized HIF1α needs to translocate into the nucleus to function as a transcription factor; therefore, we investigated the effect of SMYD3 on the nuclear HIF1α levels. Notably, overexpression of *SMYD3* enhanced HIF1α protein in the nuclei of HEK293T cells (Fig. S4*E*). In agreement, Hif1α protein level was higher in the nuclei of *Smyd3*^+/+^ MEF cells compared to the nuclei of *Smyd3*^−/−^ MEF cells, which was further confirmed by confocal microscopy (Fig. 3, *I* and *J*). Consistently, in cycloheximide pulse chase assay, overexpression of SMYD3 attenuated degradation of co-expressed HIF1α in HEK293T cells (Fig. S4*F*).

These data suggest that SMYD3 interacts with and stabilizes HIF1α, leading to an increase of nuclear HIF1α and enhanced HIF1α-mediated expression of target genes.

### The induction of HIF1α target gene expression and stabilization of HIF1α by SMYD3 are independent of HIF1α hydroxylation and pVHL intactness

**Figure 4:**
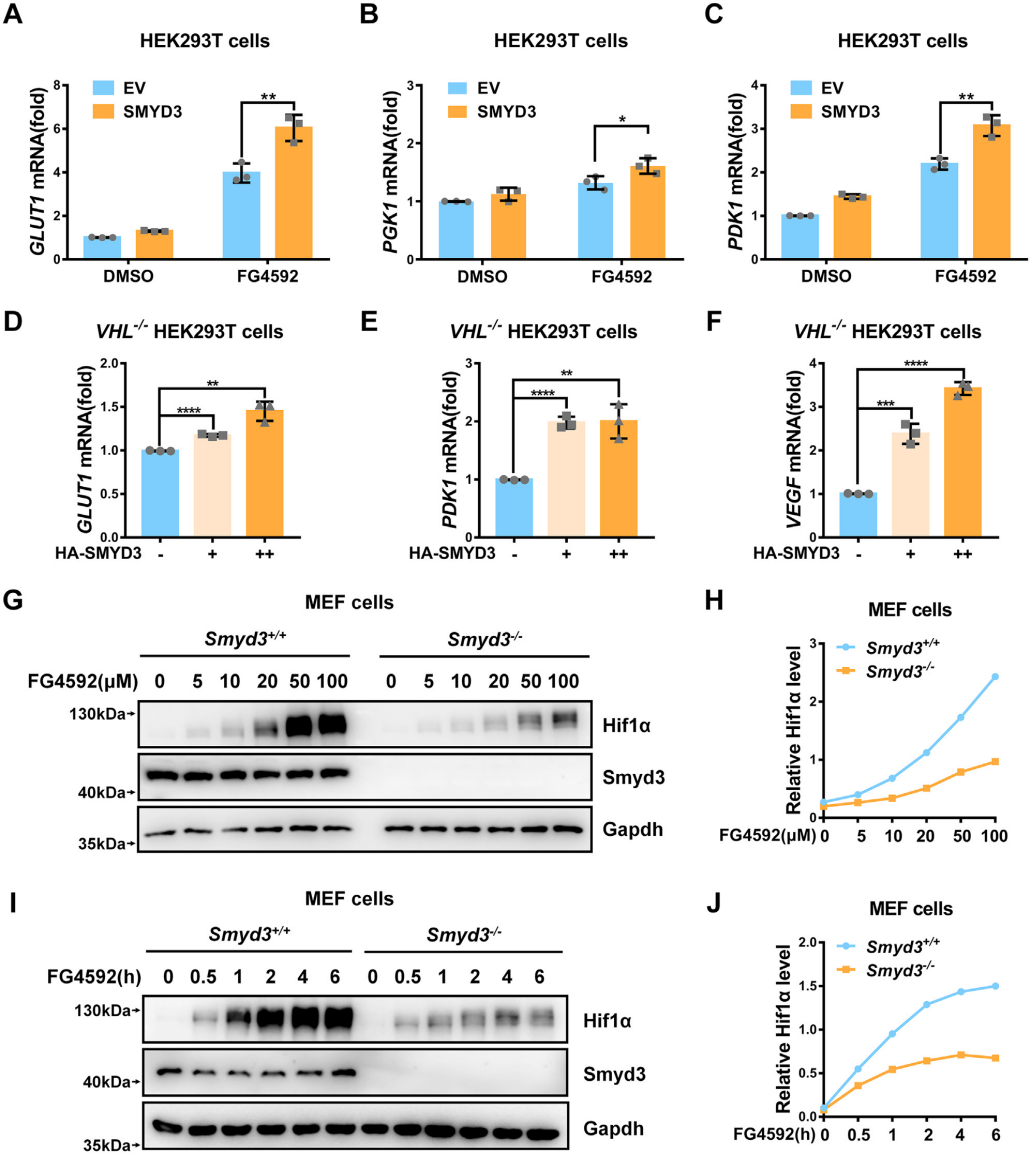
**The induction of HIF1α target genes expression and stabilization of HIF1α by SMYD3 are independent of HIF1α hydroxylation and pVHL intactness**. *A–C*, qPCR analysis of *GLUT1* (*A*), *PGK1* (*B*), and *PDK1* (*C*) mRNA in HEK293T cells transfected with or without pCMV-SMYD3 for 24 h, followed by treatment with DMSO or FG4592 (100 μM) for 8 h. EV, pCMV empty vector (control). Data show mean ± SD; Student’s two-tailed *t* test. ∗*p* < 0.05, ∗∗*p* < 0.01. Data from three independent experiments. *D–F*, qPCR analysis of *GLUT1* (*D*), *PDK1* (*E*), and *VEGF* (*F*) mRNA in *VHL*-deficient HEK293T cells (*VHL*^−/−^) transfected with an increasing amount of pCMV-SMYD3 expression plasmid. pCMV empty vector was used as a control (-). Data show mean ± SD; Student’s two-tailed *t* test. ∗∗*p* < 0.01, ∗∗∗*p* < 0.001, ∗∗∗∗*p* < 0.0001. Data from three independent experiments. *G*, immunoblotting of endogenous Hif1α expression in *Smyd3*-deficient or wildtype MEF cells (*Smyd3*^−/−^ or *Smyd3*^+/+^) treated with an increasing amount of FG4592 for 6 h. *H*, the relative intensities of Hif1α in (*G*) determined by normalizing the intensities of Hif1α to the intensities of Gapdh. *I*, immunoblotting of endogenous Hif1α expression in *Smyd3*-deficient or wildtype MEF cells (*Smyd3*^−/−^ or *Smyd3*^+/+^) treated with an increasing time of FG4592 (100 μM) for 0 to 6 h. *J*, the relative intensities of Hif1α in (*I*) determined by normalizing the intensities of Hif1α to the intensities of Gapdh. HIF, hypoxia-inducible factor; MEF, mouse embryonic fibroblast; qPCR, quantitative RT–PCR; VHL, von Hippel-Lindau.

**Supplemental Figure 5:**
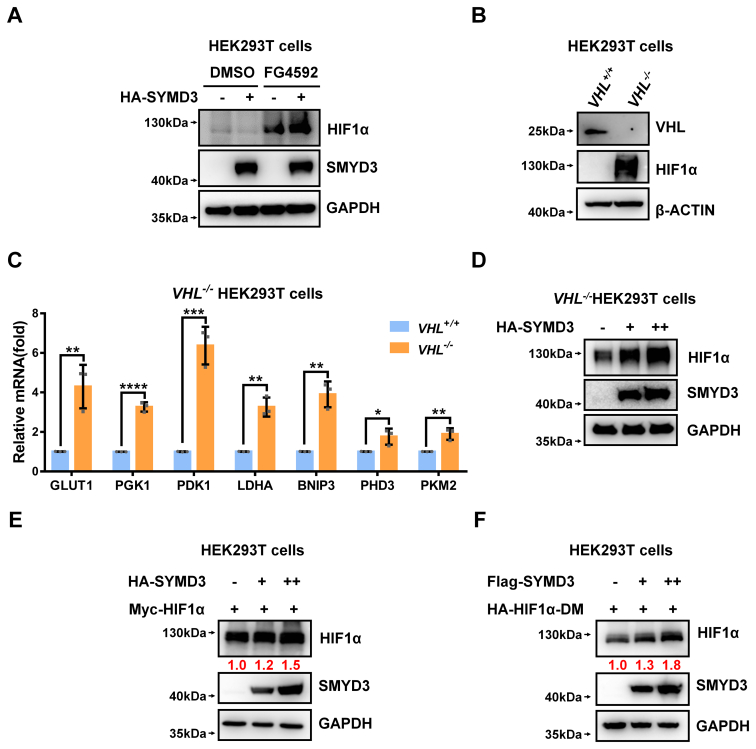


Hydroxylation of HIF1α and subsequent proteasomal degradation mediated by pVHL E3 ubiquitin ligase complex plays a central role in HIF1α regulation. We further investigated whether regulation of HIF1α by SMYD3 relies on this way. Ectopic expression of SMYD3 enhanced HIF1α protein level (Fig. S5*A*) and expression of *GLUT1*, *PGK1*, and *PDK1* induced by addition of FG4592, a specific inhibitor of PHDs (Fig. 4, *A*–*C*) (51). These data suggest that the induction of HIF1α target genes expression by *SMYD3* might not be dependent of HIF1α hydroxylation. Furthermore, we knocked out *VHL* in HEK293T cells and then examined the effect of *SMYD3* on hypoxia signaling (Fig. S5*B*). As expected, in *VHL*^-/-^ HEK293T cells, the hypoxia responsive genes, including *GLUT1*, *PGK1*, *PDK1*, *LDHA*, *BNIP3*, *PHD3*, and *PKM2*, were increased compared to those in *VHL*^+/+^ HEK293T cells (Fig. S5*C*), indicating that *VHL* was disrupted in HEK293T cells efficiently. Ectopic expression of *SMYD3* in *VHL*^-/-^ HEK293T cells enhanced HIF1α protein level (Fig. S5*D*) and hypoxia responsive gene expression (Fig. 4, *D*–*F*) in a dose-dependent manner. These data suggest that the induction of HIF1α target genes expression by *SMYD3* is independent of pVHL intactness.

In addition, co-expression of *SMYD3* together with *HIF1α* caused HIF1α protein level to increase steadily, which was not affected when the two prolyl residues (P402/P564) were mutated (HA-HIF1α-DM) (P402A/P564A) (Fig. S5, *E*–*F*). Furthermore, when FG4592 was added either in an increase of dose or in an extended time course, the protein level of endogenous Hif1α in *Smyd3*^+/+^ MEF cells kept higher than that in *Smyd3*^-/-^ MEF cells (Fig. 4, *G*–*J*).

Taken together, these data suggest that the induction of HIF1α target gene expression and stabilization of HIF1α by SMYD3 is independent of HIF1α hydroxylation and pVHL intactness.

### The stabilization and activation HIF1α by SMYD3 are independent of its methyltransferase activity

**Figure 5:**
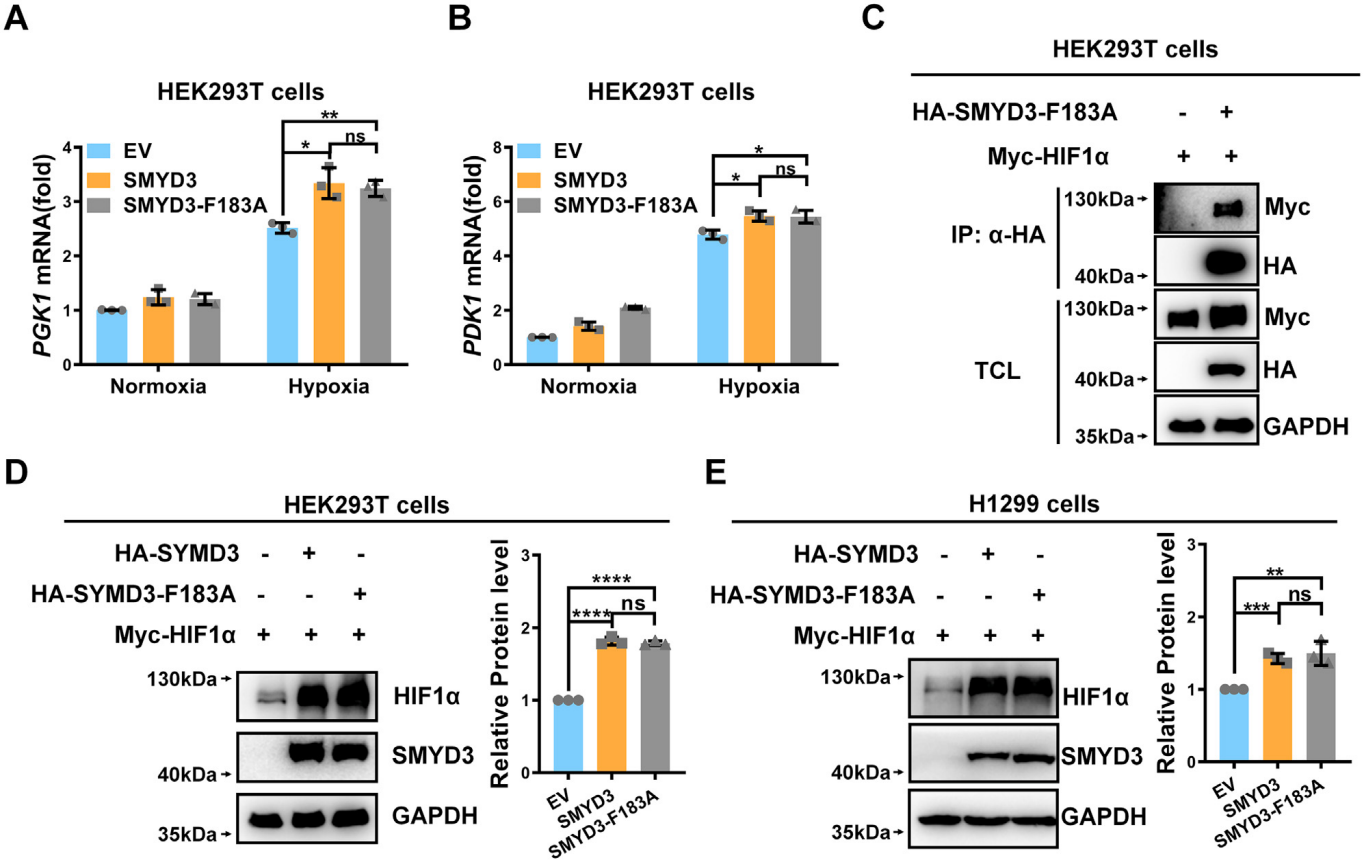
**SMYD3 stabilizes and activates HIF1α independent of its methyltransferase activity**. *A* and *B*, qPCR analysis of *PGK1* (*A*) and *PDK1* (*B*) mRNA in HEK293T cells transfected with expression plasmids encoding wildtype SMYD3 or its enzymatically dead mutant SMYD3-F183A (HA empty vector [EV] was used as a control) under normoxia (21% O_2_) or hypoxia (1% O_2_) for 24 h. Data show mean ± SD; Student’s two-tailed *t* test. ns, not significant, ∗*p* < 0.05, ∗∗*p* < 0.01. Data from three independent experiments. *C*, co-immunoprecipitation of HA-SMYD3-F183A with Myc-HIF1α. HEK293T cells were cotransfected with indicated plasmids for 24 h. Anti-HA antibody-conjugated agarose beads were used for immunoprecipitation, and the interaction was detected by immunoblotting with the indicated antibodies. *D* and *E*, immunoblotting of exogenous Myc-HIF1α expression in HEK293T (*D*) or H1299 (*E*) cells transfected with expression plasmids encoding wildtype SMYD3 or its enzymatically dead mutant SMYD3-F183A (HA empty vector [-] was used as a control). The relative intensities of HIF1α were determined by normalizing the intensities of HIF1α to the intensities of GAPDH. Data show mean ± SD; Student’s two-tailed *t* test. ns, not significant, ∗∗*p* < 0.01, ∗∗∗*p* < 0.001, ∗∗∗∗*p* < 0.0001. Data from three independent experiments. qPCR, quantitative RT–PCR; HIF, hypoxia-inducible factor.

Given that *SMYD3* serves as a methyltransferase, we sought to determine whether the modulation of HIF1α by *SMYD3* was mediated by the enzymatic activity of SMYD3. Under hypoxia, ectopic expression of enzymatic-inactive mutant of SMYD3 (SMYD3-F183A) still enhanced expression of *PGK1* and *PDK1* in HEK293T cells, similar to its wildtype form (Fig. 5, *A* and *B*).

**Supplemental Figure 6:**
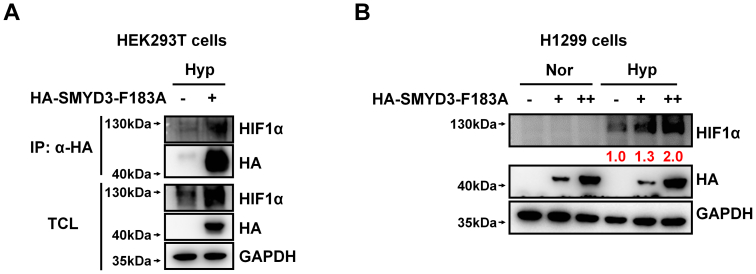


In addition, the enzymatic-inactive mutant of SMYD3 (SMYD3-F183A) still interacted with co-expressed HIF1α under normoxia (Fig. 5*C*) and endogenous HIF1α under hypoxia (Fig. S6*A*). Consistently, overexpression of SMYD3-F183A had similar effect on co-expressed HIF1α protein stability as that of wildtype SMYD3 in either HEK293T cells or H1299 cells (Fig. 5, *D* and *E*). In addition, overexpression of SMYD3-F183A still enhanced HIF1α protein stability in H1299 cells under hypoxia (Fig. S6*B*).

Taken together, these data suggest that SMYD3 stabilizes and activates HIF1α independent of its methyltransferase activity.

### SMYD3 induces ROS accumulation and enhances hypoxia-induced cell apoptosis

**Figure 6:**
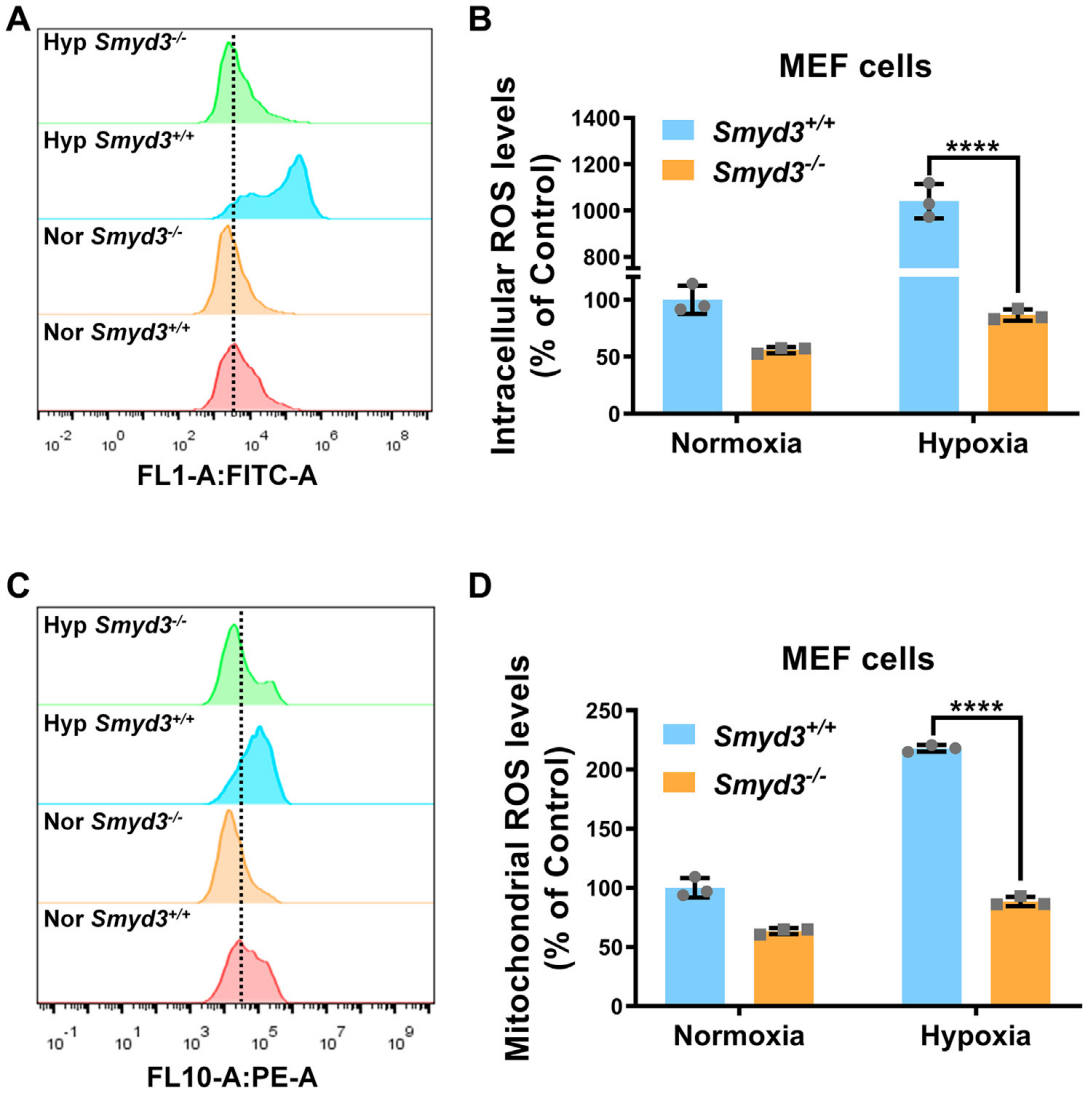
**Deficiency of SMYD3 alleviates ROS accumulation**. *A* and *B*, intracellular ROS levels in *Smyd3*-deficient or wildtype MEF cells (*Smyd3*^−/−^ or *Smyd3*^+/+^) under normoxia or hypoxia detected by flow cytometry analysis. Data show mean ± SD; Student’s two-tailed *t* test. ∗∗∗∗*p* < 0.0001. Data from three independent experiments. *C* and *D*, mitochondrial ROS levels in *Smyd3*-deficient or wildtype MEF cells (Smyd3^−/−^ or Smyd3^+/+^) under normoxia or hypoxia detected by flow cytometry analysis. Data show mean + SD; Student’s two tailed *t* test. ∗∗∗∗*p* < 0.0001. Data from three independent experiments. HIF, hypoxia-inducible factor; MEF, mouse embryonic fibroblast; ROS, reactive oxygen species.

Many studies have reported that reduction of the cytotoxic ROS level is associated with cell survival during hypoxia adaptation (52) and that aberrant control of mitochondrial ROS levels is a major factor resulting in cell apoptosis with long-term exposure to hypoxic environments (53). We examined the effect of SMYD3 on ROS accumulation. Hypoxia treatment significantly induced ROS accumulation, while much lower levels of intracellular and mitochondrial ROS were detected in *Smyd3*^−/−^ MEF cells compared to *Smyd3*^+/+^ MEF cells by flow cytometry assay (Fig. 6, *A*–*D*).

**Figure 7:**
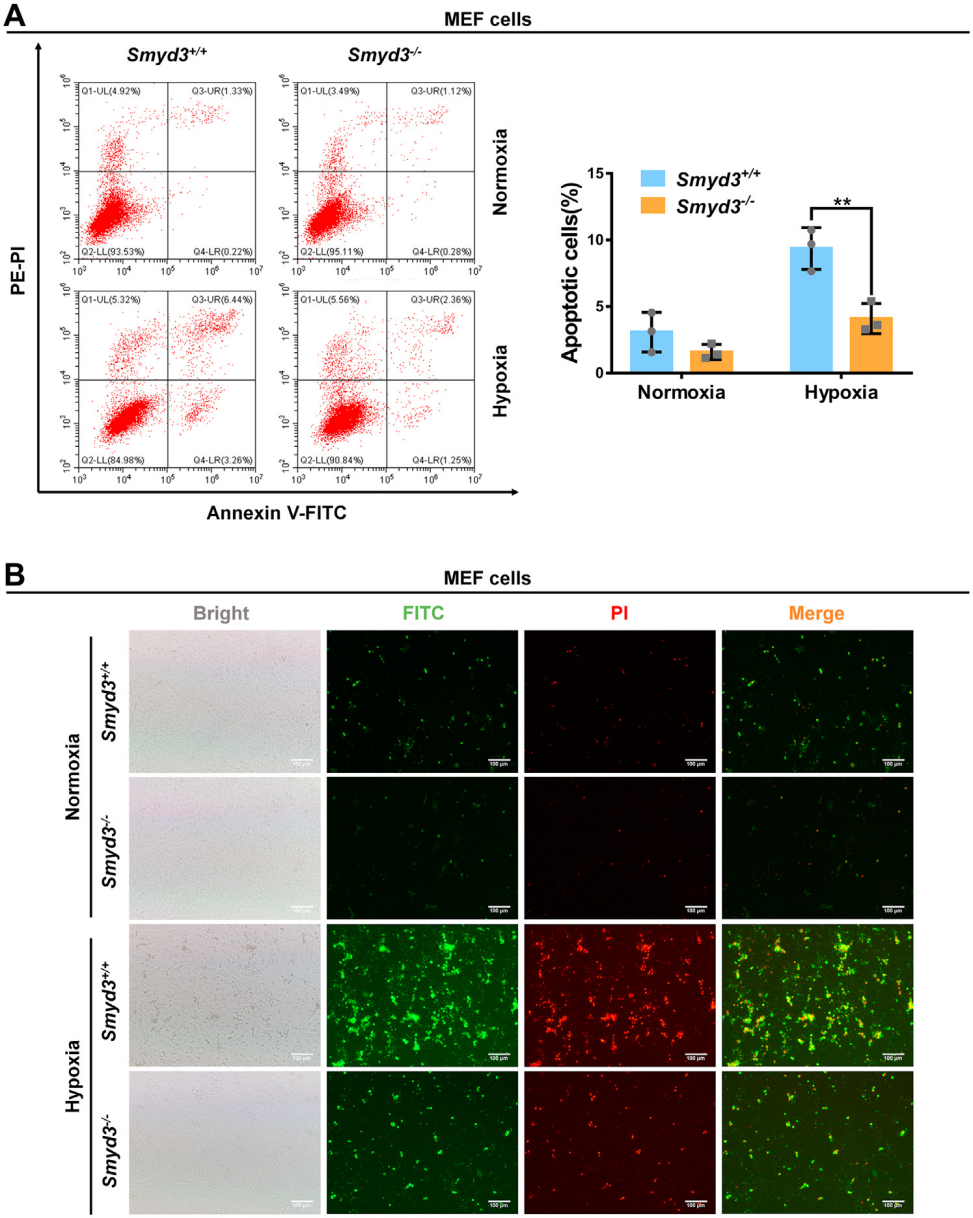
**Disruption of SMYD3 protects cells against hypoxia-induced apoptosis**. *A*, apoptotic cells in *Smyd3*-deficient or wildtype MEF cells (*Smyd3*^−/−^ or *Smyd3*^+/+^) under normoxia or hypoxia detected by flow cytometry analysis. Data show mean ± SD; Student’s two-tailed *t* test. ∗∗*p* < 0.01. Data from three independent experiments. *B*, apoptotic cells in *Smyd3*-deficient or wildtype MEF cells (*Smyd3*^−/−^ or *Smyd3*^+/+^) under normoxia or hypoxia detected by fluorescence microscopy. Scale bar = 100 μm. MEF, mouse embryonic fibroblast.

To determine the biological consequences of the transcriptional activity enhancement of HIF1α by SMYD3, we compared cell apoptosis between *Smyd3*^+/+^ and *Smyd3*^−/−^ MEF cells under hypoxia. More apoptotic cells were detected in *Smyd3*^+/+^ MEF cells compared to *Smyd3*^−/−^ MEF cells by flow cytometry assay, which was further confirmed by confocal microscopy (Fig. 7, *A* and *B*).

**Figure 8:**
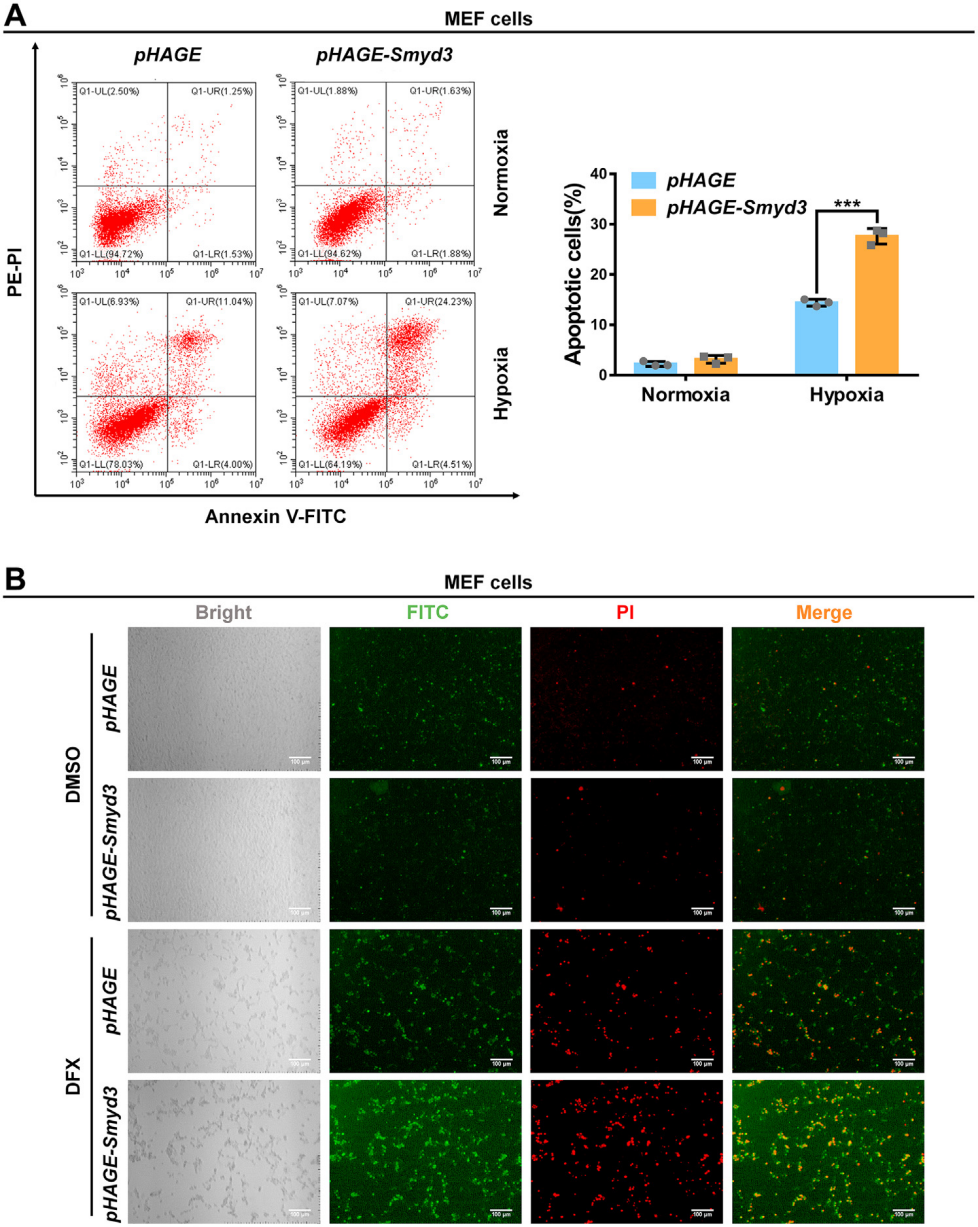
**Reconstitution of Smyd3 in *Smyd3*-deficient cells promotes hypoxia-induced apoptosis**. *A*, apoptotic cells in *Smyd3*-deficient MEF cells reconstituted with or without wildtype *Smyd3 by* lentivirus under normoxia or hypoxia detected by flow cytometry analysis. Data show mean ± SD; Student’s two-tailed *t* test. ∗∗∗*p* < 0.001. Data from three independent experiments. *B*, *Smyd3*-deficient MEF cells were reconstituted with or without wildtype *Smyd3 by* lentivirus and treated with DFX (150 μM) or DMSO as a control for 24 h. Apoptotic cells were detected by fluorescence microscopy. Scale bar = 100 μm. DFX, deferoxamine mesylate salt; MEF, mouse embryonic fibroblast.

Subsequently, we examined the effect of overexpression of *Smyd3* on cell apoptosis. In contrast, overexpression of *Smyd3* enhanced cell apoptosis under hypoxia as detected by flow cytometry assay, which was further confirmed by confocal microscopy (Fig. 8, *A* and *B*).

These data suggest that *Smyd3* enhanced hypoxia-induced apoptosis, which might be mediated by HIF1α.

### Disruption of smyd3 in zebrafish facilitates hypoxia tolerance

**Figure 9:**
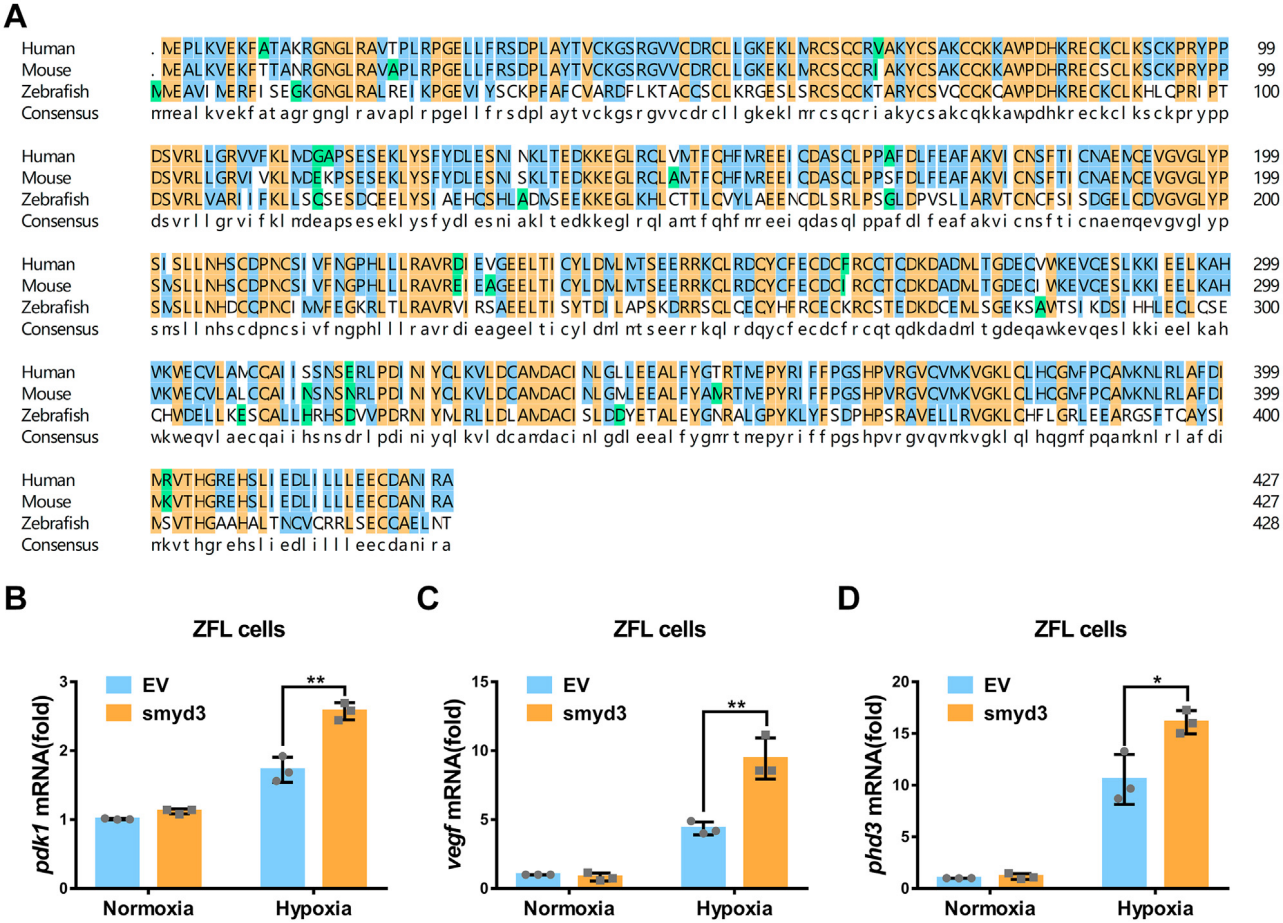
**Zebrafish smyd3 augments hypoxia signaling**. *A*, alignment of smyd3 amino acid sequences from human, mouse, and zebrafish, and the consensus sequence is shown below. *B–D*, qPCR analysis of *pdk1* (*B*), *vegf* (*C*), and *phd3* (*D*) mRNA in ZFL cells transfected with or without pCMV-smyd3 and cultured under normoxia (21% O_2_) or hypoxia (1% O_2_) for 24 h. EV, pCMV empty vector (control). Data show mean ± SD; Student’s two-tailed *t* test. ∗*p* < 0.05, ∗∗*p* < 0.01. Data from three independent experiments. qPCR, quantitative RT–PCR; ZFL, zebrafish liver.

**Figure 10:**
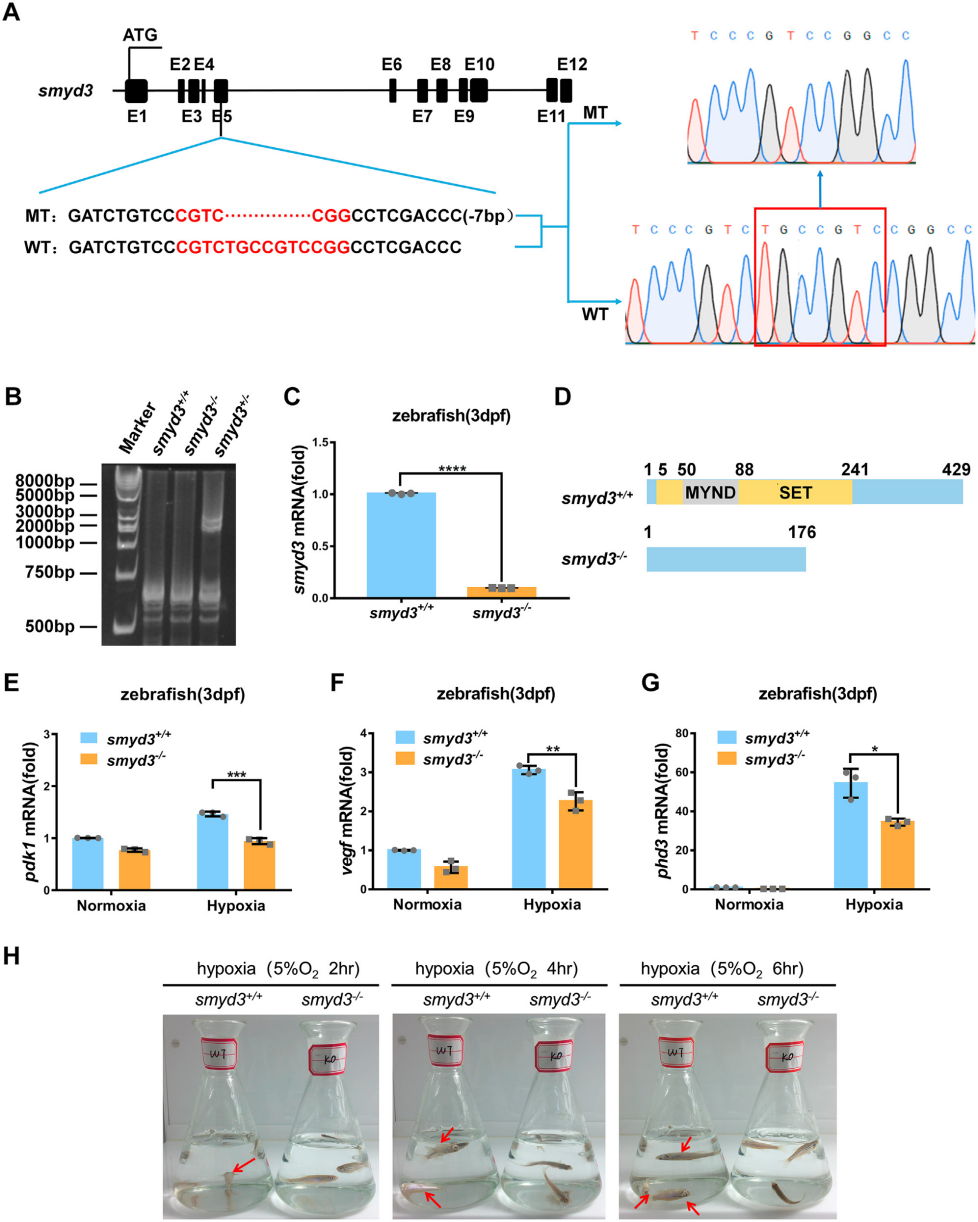
**Disruption of *smyd3* in zebrafish facilitates hypoxia tolerance**. *A*, scheme of the sequence information in *smyd3*-null zebrafish. Seven–base pair nucleotides (5′-TGCCGTC-3′) were deleted in exon five of *smyd3* in the mutant, resulting in a reading frame shift. *B*, verification of CRISPR/Cas9-mediated zebrafish *smyd3* disruption by HMA (heteroduplex mobility assay). *C*, qPCR analysis of *smyd3* mRNA in *smyd3*-deficient or wildtype zebrafish larvae (*smyd3*^−/−^ or *smyd3*^+/+^) (3dpf). Data show mean ± SD; Student’s two-tailed *t* test. ∗∗∗∗*p* < 0.0001. Data from three independent experiments. *D*, the predicted protein products of smyd3 in the mutants (176 aa) and their wildtype (429 aa) siblings. aa, amino acids. *E–G*, qPCR analysis of *pdk1* (*E*), *vegf* (*F*), and *phd3* (*G*) mRNA in *smyd3*-deficient or wildtype zebrafish larvae (*smyd3*^−/−^ or *smyd3*^+/+^) (3dpf) under normoxia (21% O_2_) or hypoxia (2% O_2_). Data show mean ± SD; Student’s two-tailed *t* test. ∗*p* < 0.05, ∗∗*p* < 0.01, ∗∗∗*p* < 0.001. Data from three independent experiments. *H*, the survival of wildtype (*smyd3*^+/+^; *left flask*) and *smyd3*-null (*smyd3*^−/−^; right flask) adult zebrafish (3mpf) after 2 h, 4 h, and 6 h under hypoxia (5% O_2_). *Red arrows*, dying zebrafish. qPCR, quantitative RT–PCR.

*SMYD3* is evolutionary conserved among human, mouse, and zebrafish (Fig. 9*A*). In zebrafish liver cells, ectopic expression of zebrafish *smyd3* caused an increase of expression of hypoxia responsive genes under hypoxia, including *pdk1*, *vegf*, and *phd3* (Fig. 9, *B*–*D*), suggesting that the function of *SMYD3* might be conserved between mammals and zebrafish. To determine the physiological role of the transcriptional activity enhancement of HIF1α by *SMYD3*, we took advantage of zebrafish model. We knocked out *smyd3* in zebrafish *via* CRISPR/Cas9 and obtained one mutant line (Fig. 10*A*). Heteroduplex mobility assay (HMA) and quantitative RT–PCR (qPCR) assay indicated that *smyd3* was disrupted efficiently in zebrafish (Fig. 10, *B* and *C*). One predicted peptide with 176 amino acids might be produced in *smyd3*-null zebrafish (Fig. 10*D*). By crossing *smyd3* ^*+/−*^ (♀) × *smyd3* ^*+/−*^ (♂), the offspring with *smyd3* ^+/+^, *smyd3* ^+/−^, and *smyd3* ^−/−^genetic backgrounds were born at a Mendelian ratio (1:2:1), and no obvious defects in growth rate and reproduction capability were detected in *smyd3* ^−/−^ zebrafish under normal conditions.

In agreement with the observations by cell culture system, under hypoxia, expression of hypoxia-responsive genes, including *pdk1*, *vegf*, and *phd3* was significantly lower in *smyd3*^−/−^ zebrafish compared to those in *smyd3* ^+/+^ zebrafish (Fig. 10, *E*–*G*). We simultaneously put *smyd3*-null zebrafish (*smyd3*^−/−^; KO) and their wildtype siblings (*smyd3*^+/+^; WT) into a hypoxia workstation (5%) and compared their hypoxia tolerance. At the beginning (1 h), no difference in behaviors was observed between *smyd3*^−/−^ and *smyd3*^+/+^ zebrafish. However, 2 h later in the hypoxia workstation (5%), *smyd3*^+/+^ zebrafish, but not *smyd3*^−/−^ zebrafish, exhibited abnormal swimming behavior (Video S1 and Fig. 10*H*, left panel). After 4 h in the hypoxia workstation (5%), *smyd3*^+/+^ zebrafish started to die (Fig. 10*H*, middle panel). After 5 to 6 h in the hypoxia workstation (5%), all of *smyd3*^+/+^ zebrafish were dead, but *smyd3*^−/−^ zebrafish were still alive (Video S2 and Fig. 10*H*, right panel). It appeared that *smyd3*^−/−^ zebrafish were more resistant to hypoxic condition.

These data suggest that *smyd3* impairs hypoxia tolerance, which might be mediated by its enhancement role on HIF1α transcriptional activity.

## Discussion

The modulation of HIF1α activity by its binding partners has been widely recognized, particularly, the most of these binding partners with enzymatic activity can regulate HIF1α activity through multiple posttranslational modifications, leading to the impacts on HIF1α activity in hypoxia signaling pathway (16, 30, 31, 45, 54, 55, 56, 57, 58). Among them, lysine methylation of HIF1α have been widely investigated. SET7-mediated monomethylation and LSD1-mediated demethylation of HIF1α at lysine 32 synergistically regulates the stability and activity of HIF1α (30, 59, 60), while monomethylation and dimethylation of HIF1α at lysine 674 by G9a/GLP inhibits its transcriptional activity and expression of its downstream target genes (61). However, whether other methyltransferases also involved in hypoxia signaling remains largely unknown. *SMYD3* is a well-defined methyltransferase (34, 35, 36). Here, we identify that SMYD3 binds to and enhances HIF1α activity, leading to the impairment of hypoxia tolerance, which is independent of its enzymatic activity. Of note, some binding partners with enzymatic activity also can affect HIF1α function independent of their enzymatic activity (31, 62, 63, 64). Therefore, it might be a common phenomenon that the proteins can affect HIF1α activity only through protein–protein interaction. However, due to the lack of structure data about the interaction between SMYD3 and HIF1α, we cannot provide more information for understanding the process and the underlying mechanisms of HIF1α activity enhancement by SMYD3.

SMYD3 contains two conserved structural domains: the Myeloid-Nervy-DEAF1 domain and the SET domain; the SET domain is consisted of the S-sequence, the core SET domain, and the C-terminus domain. The S-sequence is responsible for cofactor binding, while the core SET domain is responsible for the catalytic activity of the methyltransferase (33). Here, we find that SMYD3 binds and stabilizes HIF1α, leading to enhanced hypoxic signaling independent of its enzymatic activity. To further identify which structural domain of SMYD3 interacts HIF1α might give insights into the detailed mechanisms of SMYD3 for acting its roles in hypoxic signaling.

Given an importance of hypoxia signaling in tumor progression and cell metabolism, the present studies are mainly focused on investigating the effects of HIF1α binding partners in affecting these processes (65) (19, 58, 66, 67, 68, 69, 70, 71, 72, 73, 74, 75, 76). In fact, the roles of hypoxia signaling in hypoxia adaptation and tolerance have been noticed, particularly for high-altitude adaptation (77, 78, 79, 80, 81). High altitude is defined as areas over 2500 m above sea level, in which the ambient oxygen is much lower than low altitude area. Humans living in these areas often face great challenges due to low oxygen. Genetic evidences indicate that some human genes have gone through adaptive mutation for high altitude adaptation, and the most of them are the core components of hypoxia signaling pathway (78). In this study, by cell culture system and zebrafish model, we found that disruption of *Smyd3* impairs hypoxia-induced cell apoptosis, leading to the facilitation of hypoxia tolerance. These observations not only support an important contribution of HIF1α in hypoxia tolerance but also provide a practical research model for testing hypoxia tolerance by zebrafish model. To further use zebrafish as a model to investigate the factors involved in the regulation of hypoxia signaling as well as their impacts on hypoxia tolerance might open a new window for understanding the mechanisms of high-altitude adaptation.

In this study, we show that *SMYD3* enhances hypoxia-induced cell apoptosis, resulting in the impairment of hypoxia tolerance. However, the multiple functions of HIF1α have been identified, and *SMYD3* may also affect HIF1α functions other than hypoxia tolerance, such as tumorigenesis, cell metabolism, etc. To further figure out the other effects of SMYD3 mediated through HIF1α will help us to fully understand the physiological role of *SMYD3* in hypoxia signaling and the underlying mechanisms.

## Experimental procedures

### Cell line and culture conditions

HEK293T and H1299 cells originally obtained from American Type Culture Collection were cultured in Dulbeccos’ modified Eagle medium (VivaCell Biosciences) with 10% fetal bovine serum (FBS) at 37 °C in a humidified incubator containing 5% CO_2_. RCC4 cells were provided by Peter J. Ratcliffe and maintained as described previously (30). Zebrafish liver cells were provided by Dr Shun Li and maintained as described previously (82). *Smyd3*-deficient or wildtype MEF cells (*Smyd3*^−/−^ or *Smyd3*^+/+^) were established as described previously (83) and cultured in Dulbeccos’ modified Eagle medium supplemented with sodium pyruvate (110 mg/L), 10% FBS, 1× nonessential amino acids (Sigma), and 1% penicillin–streptomycin at 37 °C in a humidified incubator containing 5% CO_2_. During hypoxia treatment, the cells were cultured under hypoxic condition (1% O_2_, 5% CO_2_, and balanced with N_2_) by using the NBS Galaxy 48R incubator. The cells were transfected with various amounts of plasmids as indicated by VigoFect (Vigorous Biotech).

### Quantitative real-time PCR assay

Total RNAs were extracted using RNAiso Plus (TaKaRa Bio). cDNAs were synthesized using the Revert Aid First Strand cDNA Synthesis Kit (Thermo Scientific). qPCR assays were conducted with MonAmp SYBR Green qPCR Mix (high Rox) (Monad Bio.). The procedure was done according to the protocol provided by the manufacturer. The primers for quantitative RT-PCR assays are listed in Table S1.

### Antibodies and chemical reagents

Anti-SMYD3 (#ab199361) antibody was purchased from Abcam. Antibodies including anti-HIF1α (#36169), anti-VHL (#68547), anti-Histone H3 (#4499), anti-HIF2α (#7096), anti-ARNT (#5537), and normal rabbit IgG (#2729) were purchased from Cell Signaling Technology. Anti-ACTB (#AC026) antibody was purchased from ABclonal. Anti-HA (#901515) antibody was purchased from Covance. Anti-Myc (#SC-40) and anti-GAPDH (#SC-477242) antibodies were purchased from Santa Cruz Biotechnology. Anti-α-tubulin (#62204), Alexa Fluor 488 goat anti-rabbit IgG (#A11008), Alexa Fluor 594 goat anti-mouse IgG (#A11005), CM-H_2_DCFDA (#C6827), and MitoSOX Red (#M36008) were purchased from Thermo Fisher Scientific. CoCl_2_ (#C8661) and deferoxamine mesylate salt (#D9533) were purchased from Sigma. FG4592 (#S1007) and PX478 (#S7612) were purchased from Selleck. Cycloheximide (#HY-12320) was purchased from MCE.

### Immunoprecipitation and Western blot

Co-immunoprecipitation and Western blot analysis were performed as described previously (45). Anti-HA antibody-conjugated agarose beads (#A2095) were purchased from Sigma. Protein G Sepharose (#17–0618–01) was purchased from GE HealthCare Company. The blots were photographed with the Fuji Film LAS4000 mini-luminescent image analyzer. The protein levels were quantified with Image J software (National Institutes of Health) based on the band density obtained by Western blot analysis.

### CRISPR-Cas9 knockout cell lines

To generate HEK293T knocked-out cell lines of indicated genes, sgRNA sequence were ligated into Lenti-CRISPRv2 plasmid and then co-transfected with viral packaging plasmids (psPAX2 and pMD2.G) into HEK293T cells. Six hours after transfection, medium was changed, and viral supernatant was collected and filtered through 0.45-μm strainer. Targeted cells were infected by viral supernatant and selected by 1 μg/ml puromycin for 2 weeks. The sgRNA sequence targeting *VHL* was described as previously (84). The sgRNA sequence targeting *SMYD3* is 5′-CCAAGAAGTCGAACGGAGTC-3′. The sgRNA sequence targeting *ARNT* is GTCGCCGCTTAATAGCCCTC.

### Lentivirus-mediated gene transfer

HEK293T cells were transfected with pHAGE-Smyd3 or pHAGE empty vector with the packaging vectors psPAX2 and pMD2.G. Eight hours later, the medium was changed with fresh medium containing 10% FBS, 1% streptomycin–penicillin, and 10 μM β-mercaptoethanol. Forty hours later, supernatants were harvested and filtered through 0.45-μm strainer and then used to infect *Smyd3*-deficient MEF cells (*Smyd3*^−/−^).

### Immunofluorescence confocal microscopy

Immunofluorescence staining was conducted as previously described (83). Cells were seeded on glass coverslips and cultured as indicated. Then, the cells were fixed in 4% paraformaldehyde in PBS for 30 min at 25 °C. After washing three times by PBS, the slides were blocked in the blocking buffer (5% goat serum, 2 mg/ml BSA, 0.1%Triton X-100 in PBS) for 1 h and incubated with primary antibodies overnight at 4 °C, followed by incubation with Alexa Fluor 488 goat anti-rabbit IgG or Alexa Fluor 594 goat anti-mouse IgG for 1 h at 25 °C. Subsequently, the slides were mounted with VECTASHIELD mounting medium containing DAPI and photographed with Leica SP8 laser scanning confocal fluorescence microscope.

### Nucleus and cytoplasm separation

Nucleus and cytoplasm separation was conducted with the Nuclear and Cytoplasmic Extraction Kit (#78833, Thermo Scientific) according to the protocol provided by the manufacturer. The extracts were analyzed by Western blot analysis. To ensure the efficiency of fraction separation, anti-α-tubulin antibody was employed to monitor cytoplasmic proteins, and anti-Histone H3 antibody was used to monitor nuclear proteins.

### Measurement of intracellular ROS level

MEF cells were cultured under hypoxia as indicated. After treatment, MEF cells were collected and counted. Cells (1 × 10^6^) were incubated in PBS solution containing 1 μM of CM-H_2_DCFDA (#C6827, Thermo Fisher) at 37 °C for 60 min and then washed with PBS three times, followed by flow-cytometric analysis.

### Measurement of mitochondrial ROS level

MEF cells were cultured under hypoxia as indicated. After treatment, MEF cells were collected and washed with PBS. Then, the cells were incubated in PBS solution containing 5 μM of MitoSOX Red (# M36008, Thermo Fisher) for 10 min at 37 °C and then washed gently three times with PBS, followed by flow-cytometric analysis.

### Detection of apoptotic cells

MEF cells were cultured under hypoxia or treated with DFX as indicated. For flow cytometry analysis, the cells were harvested and stained with FITC-Annexin V and PI with FITC Annexin V Apoptosis Detection Kit I (#556547, BD Pharmingen) according to the manufacturer’s instructions. Apoptotic cells were detected using Beckman CytoFLEXS, and the data were analyzed with CytExpert software. Besides, the cells were stained with Annexin V-FITC Apoptosis Detection Kit (#C1062, Beyotime) according to the manufacturer’s instructions in 6-well plate and imaged under a florescent microscope Nikon TE2000-U.

### Generation of smyd3-null zebrafish

Disruption of *smyd3* in zebrafish was accomplished *via* CRISPR/Cas9 technology. Zebrafish *smyd3* sgRNA was designed using the tools provided in the CRISPR Design web site (http://crispr.mit.edu). The sgRNA sequence targeting *smyd3* is 5′-TCTGCCGTCCGGCCTCGAC-3′ and sgRNA was synthesized using the Transcript Aid T7 High Yield Transcription Kit (Fermentas). Cas9 RNA and sgRNA were prepared as described previously (82) and then mixed and injected into embryos at the one-cell stage. Mutant detection was followed by HMA as described previously (30). If the results were positive, the remaining embryos were raised to adulthood and treated as F0. The F0 zebrafish were backcrossed with the wildtype zebrafish to generate F1, which were genotyped by HMA and then confirmed by sequencing of target sites. The F1 zebrafish harboring the mutations were backcrossed with the wild-type zebrafish to obtain F2. The F2 adult zebrafish with the same genotype (+/−) were intercrossed to generate F3 offspring, which should contain wild-type (+/+), heterozygous (+/−), and homozygous (−/−) offspring. The primers for detecting mutants were 5′-ATCTCGCAGACATGAGTGAG-3’ (forward) and 5′-CACCGGTCTGACAGCAGCAG-3’ (reverse). The zebrafish *smyd3* mutant was named *smyd3*^ihbsm3^/^ihbsm3^ (https://zfin.org/ZDB-ALT-220302-1) following zebrafish nomenclature guidelines (zfin.atlassian.net/wiki/spaces/general/pages/1818394635/ZFIN+Zebrafish+Nomenclature+Conventions).

### Hypoxia treatments of zebrafish

Hypoxia treatments of zebrafish were conducted in the hypoxia workstation (Ruskinn INVIVO2 I-400) as described previously (85). For zebrafish larvae (3 days postfertilization [dpf]) experiment, two dish were filled with 10 ml of water. *Smyd3*-null larvae (3 dpf, n = 30) (*smyd3*^−/−^) were put into one dish, and their wildtype siblings (3 dpf, n = 30) (*smyd3*^+/+^) were put in the second dish. The oxygen concentration in the hypoxia workstation was adjusted to 2% ahead of time. Then, two dishes were put into the hypoxia workstation simultaneously. Four hours later, the samples were harvested for qPCR analysis. This experiment was repeated three times. For the adult zebrafish (3-months postfertilization [mpf]) experiment, zebrafish of similar weight were chosen for further experiments. Two flasks were filled with 200 ml of water. Three *smyd3*-null zebrafish (*smyd3*^−/−^) were put into one flask, and three wildtype siblings (*smyd3*^+/+^) were put into the second flask. The oxygen concentration in the hypoxia workstation was adjusted to 5% ahead of time. After putting the flasks containing zebrafish into the hypoxia workstation, the behavior of the zebrafish was closely monitored. All animal protocols were approved by the Institutional Animal Care and Use Committee at Institute of Hydrobiology, Chinese Academy of Science.

### Statical analysis

GraphPad Prism software (7.0) was used for all statistical analysis. Results with error bars express mean ± SD. Statistical analysis was performed by using Student’s two-tailed *t* test. A *p* value less than 0.05 was considered significant. Statistical significance is represented as follows: ∗*p*< 0.05, ∗∗*p* < 0.01, ∗∗∗*p* < 0.001, ∗∗∗∗*p* < 0.0001.

## Data availability

Further information and requests for resources and reagents should be directed to and will be fulfilled by Xing Liu and Wuhan Xiao.

## Supporting information

This article contains supporting information.

## Conflict of interest

The authors declare that they have no conflicts of interest with the contents of this article.

## References

[1] Lee P., Chandel N.S., Simon M.C. (2020). Cellular adaptation to hypoxia through hypoxia inducible factors and beyond. Nat. Rev. Mol. Cell Biol..

[2] Kim J.W., Tchernyshyov I., Semenza G.L., Dang C.V. (2006). HIF-1-mediated expression of pyruvate dehydrogenase kinase: a metabolic switch required for cellular adaptation to hypoxia. Cell Metab..

[3] Fuhrmann D.C., Brune B. (2017). Mitochondrial composition and function under the control of hypoxia. Redox Biol..

[4] Sies H., Belousov V.V., Chandel N.S., Davies M.J., Jones D.P., Mann G.E. (2022). Defining roles of specific reactive oxygen species (ROS) in cell biology and physiology. Nat. Rev. Mol. Cell Biol..

[5] Smith K.A., Waypa G.B., Schumacker P.T. (2017). Redox signaling during hypoxia in mammalian cells. Redox Biol..

[6] Kaelin W.G., Ratcliffe P.J. (2008). Oxygen sensing by metazoans: the central role of the HIF hydroxylase pathway. Mol. Cell.

[7] Majmundar A.J., Wong W.J., Simon M.C. (2010). Hypoxia-inducible factors and the response to hypoxic stress. Mol. Cell.

[8] Ivan M., Kaelin W.G. (2017). The EGLN-HIF O2-sensing system: multiple inputs and feedbacks. Mol. Cell.

[9] Semenza G.L. (2009). Regulation of oxygen homeostasis by hypoxia-inducible factor 1. Physiology (Bethesda).

[10] Semenza G.L. (2014). Oxygen sensing, hypoxia-inducible factors, and disease pathophysiology. Annu. Rev. Pathol..

[11] Hammarlund E.U., Flashman E., Mohlin S., Licausi F. (2020). Oxygen-sensing mechanisms across eukaryotic kingdoms and their roles in complex multicellularity. Science.

[12] Kaelin W.G. (2005). Proline hydroxylation and gene expression. Annu. Rev. Biochem..

[13] Schofield C.J., Ratcliffe P.J. (2004). Oxygen sensing by HIF hydroxylases. Nat. Rev. Mol. Cell Biol..

[14] Isaacs J.S., Jung Y.J., Mole D.R., Lee S., Torres-Cabala C., Chung Y.L. (2005). HIF overexpression correlates with biallelic loss of fumarate hydratase in renal cancer: novel role of fumarate in regulation of HIF stability. Cancer Cell.

[15] Kong X., Lin Z., Liang D., Fath D., Sang N., Caro J. (2006). Histone deacetylase inhibitors induce VHL and ubiquitin-independent proteasomal degradation of hypoxia-inducible factor 1alpha. Mol. Cell Biol..

[16] Kubaichuk K., Kietzmann T. (2019). Involvement of E3 ligases and deubiquitinases in the control of HIF-alpha subunit abundance. Cells.

[17] Mahon P.C., Hirota K., Semenza G.L. (2001). FIH-1: a novel protein that interacts with HIF-1alpha and VHL to mediate repression of HIF-1 transcriptional activity. Genes Dev..

[18] Albanese A., Daly L.A., Mennerich D., Kietzmann T., See V. (2020). The role of hypoxia-inducible factor post-translational modifications in regulating its localisation, stability, and activity. Int. J. Mol. Sci..

[19] Lim J.H., Lee Y.M., Chun Y.S., Chen J., Kim J.E., Park J.W. (2010). Sirtuin 1 modulates cellular responses to hypoxia by deacetylating hypoxia-inducible factor 1alpha. Mol. Cell.

[20] Dioum E.M., Chen R., Alexander M.S., Zhang Q., Hogg R.T., Gerard R.D. (2009). Regulation of hypoxia-inducible factor 2alpha signaling by the stress-responsive deacetylase sirtuin 1. Science.

[21] Bae S.H., Jeong J.W., Park J.A., Kim S.H., Bae M.K., Choi S.J. (2004). Sumoylation increases HIF-1alpha stability and its transcriptional activity. Biochem. Biophys. Res. Commun..

[22] Berta M.A., Mazure N., Hattab M., Pouyssegur J., Brahimi-Horn M.C. (2007). SUMOylation of hypoxia-inducible factor-1alpha reduces its transcriptional activity. Biochem. Biophys. Res. Commun..

[23] Cheng J., Kang X., Zhang S., Yeh E.T. (2007). SUMO-specific protease 1 is essential for stabilization of HIF1alpha during hypoxia. Cell.

[24] Carbia-Nagashima A., Gerez J., Perez-Castro C., Paez-Pereda M., Silberstein S., Stalla G.K. (2007). RSUME, a small RWD-containing protein, enhances SUMO conjugation and stabilizes HIF-1alpha during hypoxia. Cell.

[25] Mylonis I., Chachami G., Samiotaki M., Panayotou G., Paraskeva E., Kalousi A. (2006). Identification of MAPK phosphorylation sites and their role in the localization and activity of hypoxia-inducible factor-1alpha. J. Biol. Chem..

[26] Warfel N.A., Dolloff N.G., Dicker D.T., Malysz J., El-Deiry W.S. (2013). CDK1 stabilizes HIF-1alpha *via* direct phosphorylation of Ser668 to promote tumor growth. Cell Cycle.

[27] Kalousi A., Mylonis I., Politou A.S., Chachami G., Paraskeva E., Simos G. (2010). Casein kinase 1 regulates human hypoxia-inducible factor HIF-1. J. Cell Sci..

[28] Xu D., Yao Y., Lu L., Costa M., Dai W. (2010). Plk3 functions as an essential component of the hypoxia regulatory pathway by direct phosphorylation of HIF-1alpha. J. Biol. Chem..

[29] Geng H., Harvey C.T., Pittsenbarger J., Liu Q., Beer T.M., Xue C. (2011). HDAC4 protein regulates HIF1alpha protein lysine acetylation and cancer cell response to hypoxia. J. Biol. Chem..

[30] Liu X., Chen Z., Xu C., Leng X., Cao H., Ouyang G. (2015). Repression of hypoxia-inducible factor alpha signaling by Set7-mediated methylation. Nucl. Acids Res..

[31] Wang J., Zhang D., Du J., Zhou C., Li Z., Liu X. (2017). Tet1 facilitates hypoxia tolerance by stabilizing the HIF-alpha proteins independent of its methylcytosine dioxygenase activity. Nucl. Acids Res..

[32] Semenza G.L. (2017). A compendium of proteins that interact with HIF-1alpha. Exp. Cell Res..

[33] Tracy C., Warren J.S., Szulik M., Wang L., Garcia J., Makaju A. (2018). The smyd family of methyltransferases: role in cardiac and skeletal muscle physiology and pathology. Curr. Opin. Physiol..

[34] Bottino C., Peserico A., Simone C., Caretti G. (2020). SMYD3: an oncogenic driver targeting epigenetic regulation and signaling pathways. Cancers (Basel).

[35] Bernard B.J., Nigam N., Burkitt K., Saloura V. (2021). SMYD3: A regulator of epigenetic and signaling pathways in cancer. Clin. Epigenetics.

[36] Hamamoto R., Furukawa Y., Morita M., Iimura Y., Silva F.P., Li M. (2004). SMYD3 encodes a histone methyltransferase involved in the proliferation of cancer cells. Nat. Cell Biol..

[37] Van Aller G.S., Reynoird N., Barbash O., Huddleston M., Liu S., Zmoos A.F. (2012). Smyd3 regulates cancer cell phenotypes and catalyzes histone H4 lysine 5 methylation. Epigenetics.

[38] Kunizaki M., Hamamoto R., Silva F.P., Yamaguchi K., Nagayasu T., Shibuya M. (2007). The lysine 831 of vascular endothelial growth factor receptor 1 is a novel target of methylation by SMYD3. Cancer Res..

[39] Mazur P.K., Reynoird N., Khatri P., Jansen P.W., Wilkinson A.W., Liu S. (2014). SMYD3 links lysine methylation of MAP3K2 to Ras-driven cancer. Nature.

[40] Yoshioka Y., Suzuki T., Matsuo Y., Nakakido M., Tsurita G., Simone C. (2016). SMYD3-mediated lysine methylation in the PH domain is critical for activation of AKT1. Oncotarget.

[41] Kim H., Heo K., Kim J.H., Kim K., Choi J., An W. (2009). Requirement of histone methyltransferase SMYD3 for estrogen receptor-mediated transcription. J. Biol. Chem..

[42] Yoshioka Y., Suzuki T., Matsuo Y., Tsurita G., Watanabe T., Dohmae N. (2017). Protein lysine methyltransferase SMYD3 is involved in tumorigenesis through regulation of HER2 homodimerization. Cancer Med..

[43] Sarris M.E., Moulos P., Haroniti A., Giakountis A., Talianidis I. (2016). Smyd3 is a transcriptional potentiator of multiple cancer-promoting genes and required for liver and colon cancer development. Cancer Cell.

[44] Mazur P.K., Gozani O., Sage J., Reynoird N. (2016). Novel insights into the oncogenic function of the SMYD3 lysine methyltransferase. Transl Cancer Res..

[45] Chen Z., Liu X., Mei Z., Wang Z., Xiao W. (2014). EAF2 suppresses hypoxia-induced factor 1alpha transcriptional activity by disrupting its interaction with coactivator CBP/p300. Mol. Cell Biol..

[46] Xu C., Liu X., Zha H., Fan S., Zhang D., Li S. (2018). A pathogen-derived effector modulates host glucose metabolism by arginine GlcNAcylation of HIF-1alpha protein. PLoS Pathog..

[47] Wang G.L., Semenza G.L. (1993). Desferrioxamine induces erythropoietin gene expression and hypoxia-inducible factor 1 DNA-binding activity: implications for models of hypoxia signal transduction. Blood.

[48] Chandel N.S., Maltepe E., Goldwasser E., Mathieu C.E., Simon M.C., Schumacker P.T. (1998). Mitochondrial reactive oxygen species trigger hypoxia-induced transcription. Proc. Natl. Acad. Sci. U. S. A..

[49] Zhang L., Jin Y., Yang H., Li Y., Wang C., Shi Y. (2019). SMYD3 promotes epithelial ovarian cancer metastasis by downregulating p53 protein stability and promoting p53 ubiquitination. Carcinogenesis.

[50] Zhang C., Liu J., Wang J., Zhang T., Xu D., Hu W. (2021). The interplay between tumor suppressor p53 and hypoxia signaling pathways in cancer. Front. Cell Dev. Biol..

[51] Rabinowitz M.H. (2013). Inhibition of hypoxia-inducible factor prolyl hydroxylase domain oxygen sensors: tricking the body into mounting orchestrated survival and repair responses. J. Med. Chem..

[52] Kim T.H., Hur E.G., Kang S.J., Kim J.A., Thapa D., Lee Y.M. (2011). NRF2 blockade suppresses colon tumor angiogenesis by inhibiting hypoxia-induced activation of HIF-1alpha. Cancer Res..

[53] Lee H.J., Jung Y.H., Choi G.E., Ko S.H., Lee S.J., Lee S.H. (2017). BNIP3 induction by hypoxia stimulates FASN-dependent free fatty acid production enhancing therapeutic potential of umbilical cord blood-derived human mesenchymal stem cells. Redox Biol..

[54] Goto Y., Zeng L., Yeom C.J., Zhu Y., Morinibu A., Shinomiya K. (2015). UCHL1 provides diagnostic and antimetastatic strategies due to its deubiquitinating effect on HIF-1alpha. Nat. Commun..

[55] Hong K., Hu L., Liu X., Simon J.M., Ptacek T.S., Zheng X. (2020). USP37 promotes deubiquitination of HIF2alpha in kidney cancer. Proc. Natl. Acad. Sci. U. S. A..

[56] Troilo A., Alexander I., Muehl S., Jaramillo D., Knobeloch K.P., Krek W. (2014). HIF1alpha deubiquitination by USP8 is essential for ciliogenesis in normoxia. EMBO Rep..

[57] Wu H.T., Kuo Y.C., Hung J.J., Huang C.H., Chen W.Y., Chou T.Y. (2016). K63-polyubiquitinated HAUSP deubiquitinates HIF-1alpha and dictates H3K56 acetylation promoting hypoxia-induced tumour progression. Nat. Commun..

[58] Shay J.E.S., Simon M.C. (2012). Hypoxia-inducible factors: crosstalk between inflammation and metabolism. Semin. Cell Dev. Biol..

[59] Kim Y., Nam H.J., Lee J., Park D.Y., Kim C., Yu Y.S. (2016). Methylation-dependent regulation of HIF-1alpha stability restricts retinal and tumour angiogenesis. Nat. Commun..

[60] Lee J.Y., Park J.H., Choi H.J., Won H.Y., Joo H.S., Shin D.H. (2017). LSD1 demethylates HIF1alpha to inhibit hydroxylation and ubiquitin-mediated degradation in tumor angiogenesis. Oncogene.

[61] Bao L., Chen Y., Lai H.T., Wu S.Y., Wang J.E., Hatanpaa K.J. (2018). Methylation of hypoxia-inducible factor (HIF)-1alpha by G9a/GLP inhibits HIF-1 transcriptional activity and cell migration. Nucl. Acids Res..

[62] Li B., Qiu B., Lee D.S.M., Walton Z.E., Ochocki J.D., Mathew L.K. (2014). Fructose-1,6-bisphosphatase opposes renal carcinoma progression. Nature.

[63] Hubbi M.E., Hu H.X., Kshitiz, Gilkes D.M., Semenza G.L. (2013). Sirtuin-7 inhibits the activity of hypoxia-inducible factors. J. Biol. Chem..

[64] Altun M., Zhao B., Velasco K., Liu H.Y., Hassink G., Paschke J. (2012). Ubiquitin-specific protease 19 (USP19) regulates hypoxia-inducible factor 1 alpha (HIF-1 alpha) during hypoxia. J. Biol. Chem..

[65] Luo W., Hu H., Chang R., Zhong J., Knabel M., O'Meally R. (2011). Pyruvate kinase M2 is a PHD3-stimulated coactivator for hypoxia-inducible factor 1. Cell.

[66] Zhang X., Wang K., Feng X., Wang J., Chu Y., Jia C. (2021). PRMT3 promotes tumorigenesis by methylating and stabilizing HIF1alpha in colorectal cancer. Cell Death Dis..

[67] Tang X., Chang C., Hao M., Chen M., Woodley D.T., Schonthal A.H. (2021). Heat shock protein-90alpha (Hsp90alpha) stabilizes hypoxia-inducible factor-1alpha (HIF-1alpha) in support of spermatogenesis and tumorigenesis. Cancer Gene Ther..

[68] Fang W.T., Liao C.H., Shi R., Simon J.M., Ptacek T.S., Zurlo G. (2021). ZHX2 promotes HIF1 alpha oncogenic signaling in triple-negative breast cancer. Elife.

[69] Shao A.W., Lang Y., Wang M.D., Qin C., Kuang Y., Mei Y.D. (2020). Bclaf1 is a direct target of HIF-1 and critically regulates the stability of HIF-1 alpha under hypoxia. Oncogene.

[70] Rezaeian A.H., Li C.F., Wu C.Y., Zhang X., Delacerda J., You M.J. (2017). A hypoxia-responsive TRAF6-ATM-H2AX signalling axis promotes HIF1 alpha activation, tumorigenesis and metastasis. Nat. Cell Biol..

[71] Semenza G.L. (2003). Targeting HIF-1 for cancer therapy. Nat. Rev. Cancer.

[72] Kierans S.J., Taylor C.T. (2021). Regulation of glycolysis by the hypoxia-inducible factor (HIF): implications for cellular physiology. J. Physiol..

[73] Zhong L., D'Urso A., Toiber D., Sebastian C., Henry R.E., Vadysirisack D.D. (2010). The histone deacetylase Sirt6 regulates glucose homeostasis *via* Hif1alpha. Cell.

[74] Finley L.W., Carracedo A., Lee J., Souza A., Egia A., Zhang J. (2011). SIRT3 opposes reprogramming of cancer cell metabolism through HIF1alpha destabilization. Cancer Cell.

[75] Gunton J.E. (2020). Hypoxia-inducible factors and diabetes. J. Clin. Invest.

[76] Gonzalez F.J., Xie C., Jiang C. (2018). The role of hypoxia-inducible factors in metabolic diseases. Nat. Rev. Endocrinol..

[77] Bigham A.W., Lee F.S. (2014). Human high-altitude adaptation: forward genetics meets the HIF pathway. Gene Dev..

[78] Bigham A.W. (2016). Genetics of human origin and evolution: high-altitude adaptations. Curr. Opin. Genet. Dev..

[79] Huerta-Sanchez E., Jin X., Asan, Bianba Z., Peter B.M., Vinckenbosch N., Liang Y. (2014). Altitude adaptation in Tibetans caused by introgression of Denisovan-like DNA. Nature.

[80] Jeong C.W., Alkorta-Aranburu G., Basnyat B., Neupane M., Witonsky D.B., Pritchard J.K. (2014). Admixture facilitates genetic adaptations to high altitude in Tibet. Nat. Commun..

[81] Lorenzo F.R., Huff C., Myllymaki M., Olenchock B., Swierczek S., Tashi T. (2014). A genetic mechanism for Tibetan high-altitude adaptation. Nat. Genet..

[82] Wu H., Sun L., Wen Y., Liu Y., Yu J., Mao F. (2016). Major spliceosome defects cause male infertility and are associated with nonobstructive azoospermia in humans. Proc. Natl. Acad. Sci. U. S. A..

[83] Liu X., Zhu C., Zha H., Tang J., Rong F., Chen X. (2020). SIRT5 impairs aggregation and activation of the signaling adaptor MAVS through catalyzing lysine desuccinylation. EMBO J..

[84] Zhang J., Wu T., Simon J., Takada M., Saito R., Fan C. (2018). VHL substrate transcription factor ZHX2 as an oncogenic driver in clear cell renal cell carcinoma. Science.

[85] Liu X., Cai X., Hu B., Mei Z., Zhang D., Ouyang G. (2016). Forkhead transcription factor 3a (FOXO3a) modulates hypoxia signaling *via* up-regulation of the von Hippel-lindau gene (VHL). J. Biol. Chem..

