# Supplementary material for: Methyltransferase SMYD3 impairs hypoxia tolerance by augmenting hypoxia signaling independent of its enzymatic activity
Source: J Biol Chem. 2022 Oct 21;298(12):102633. doi: 10.1016/j.jbc.2022.102633 (PMC9692045; doi:10.1016/j.jbc.2022.102633)
Supplement: Supplementary information [file mmc1.docx]

**Supplementary information**

**Supplementary Data**

**Figure S1.** **Hypoxia suppresses expression of SMYD3, and SMYD3 augments hypoxia signaling in H1299 cells.**

(A) qPCR analysis of *SMYD3* mRNA in H1299 cells cultured under normoxia (21% O_2_) or hypoxia (1% O_2_) for 24 h.

(B) Immunoblotting of indicated proteins in *ARNT*-deficient or wildtype H1299 cells (*ARNT*^-/-^ or *ARNT*^+/+^).

(C) qPCR analysis of *PGK1* mRNA in *ARNT*-deficient or wildtype H1299 cells (*ARNT*^-/-^ or *ARNT*^+/+^) cultured under normoxia (21% O_2_) or hypoxia (1% O_2_) for 24 h.

(D) qPCR analysis of *SMYD3* mRNA in *ARNT*-deficient H1299 cells (*ARNT*^-/-^) cultured under normoxia (21% O_2_) or hypoxia (1% O_2_) for 24 h.

(E) qPCR analysis of *PGK1* mRNA in H1299 cells treated with an increasing amount of PX478 (0-100 μM) and cultured under normoxia (21% O_2_) or hypoxia (1% O_2_) for 24 h.

(F) qPCR analysis of *SMYD3* mRNA in H1299 cells treated with PX478 (100 μM) and cultured under normoxia (21% O_2_) or hypoxia (1% O_2_) for 24 h.

(G-I) qPCR analysis of *GLUT1* (G), *PGK1* (H) and *VEGF* (I) mRNA in H1299 cells transfected with or without pCMV-SMYD3 and cultured under normoxia (21% O_2_) or hypoxia (1% O_2_) for 24 h. EV, pCMV empty vector (control).

Data show mean + SD; Student’s two tailed t-test. *p<0.05, **p<0.01, ***p<0.001, ****p<0.0001. Data from three independent experiments.

**Figure S2. SMYD3 deficiency suppresses hypoxia signaling.**

(A) Immunoblotting of indicated proteins in Figure 2B and 2C.

(B) Immunoblotting of indicated proteins in Figure 2D to 2F.

(C) Immunoblotting of indicated proteins in Figure 2H and 2I.

(D) Immunoblotting of indicated proteins in Figure 2K and 2L.

(E) qPCR analysis of *SMYD3* mRNA in HEK293T cells transfected with control shRNA, *SMYD3*-shRNA#1 or *SMYD3*-shRNA#2.

(F-H) qPCR analysis of *GLUT1* (F), *PDK1* (G), and *PGK1* (H) mRNA in HEK293T cells transfected with control shRNA, *SMYD3*-shRNA#1 or *SMYD3*-shRNA#2, and then cultured under normoxia (21% O_2_) or hypoxia (1% O_2_) for another 24 h.

Data show mean + SD; Student’s two tailed t-test. ***p<0.001, ****p<0.0001. Data from three independent experiments.

**Figure S3. SMYD3 has similar effect on HIF2α as that on HIF1α.**

(A) qPCR analysis of *CITED2* mRNA in HEK293T cells transfected with or without pCMV-SMYD3 under normoxia (21% O_2_) or hypoxia (1% O_2_) for 24 h. Data show mean + SD; Student’s two tailed t-test. ***p<0.001. Data from three independent experiments.

(B) qPCR analysis of *CITED2* mRNA in *SMYD3*-deficient or wildtype HEK293T cells (*SMYD3*^-/-^ or *SMYD3*^+/+^) under normoxia (21% O_2_) or hypoxia (1% O_2_) for 24 h. Data show mean + SD; Student’s two tailed t-test. **p<0.01. Data from three independent experiments.

(C) qPCR analysis of *CITED2* mRNA in *SMYD3*-deficient or wildtype HEK293T cells (*SMYD3*^-/-^ or *SMYD3*^+/+^) treated with or without CoCl_2_ (200 μM) for 8 h. Data show mean + SD; Student’s two tailed t-test. ***p<0.001. Data from three independent experiments.

(D) Immunoblotting of indicated proteins in Figure S3A.

(E) Immunoblotting of indicated proteins in Figure S3B.

(F) Immunoblotting of indicated proteins in Figure S3C.

**Figure S4. SMYD3 binds to and stabilizes HIF1α, leading to an increase of nuclear HIF1α.**

(A) Immunoblotting of indicated proteins in Figure 3A to 3C.

(B) Co-immunoprecipitation of exogenous HA-SMYD3 with endogenous HIF1α. HEK293T cells were transfected with HA-SMYD3 or pCMV-HA empty vector for 24 h and then cultured hypoxia (1% O_2_) for 4 h. Anti-HA antibody-conjugated agarose beads were used for immunoprecipitation, and the interaction was detected by immunoblotting with the indicated antibodies.

(C) Endogenous interaction between SMYD3 and HIF1α. HEK293T cells were cultured under hypoxia (1% O_2_) for 4 h and anti-HIF1α antibody or normal rabbit IgG was used for immunoprecipitation.

(D) Immunoblotting of endogenous HIF1α expression in H1299 cells transfected with an increasing amount of HA-SMYD3 expression plasmid (HA empty vector [-] was used as a control) for 24 h, and then cultured under normoxia (21% O_2_) or hypoxia (1% O_2_) for 4 h.

(E) Confocal microscopy image of endogenous HIF1α in HEK293T cells transfected with or without HA-SMYD3 under hypoxia for 4 h. Scale bar = 25 μm.

(F) Immunoblotting of indicated proteins in HEK293T cells transfected with Myc-HIF1α together with HA-SMYD3 or pCMV-HA empty vector for 24 h and then treated with an increasing time (0-6 h) of CHX (50 μg/ml).

**Figure S5. Stabilization of HIF1α by SMYD3 is independent of HIF1α hydroxylation and pVHL intactness.**

(A) Immunoblotting of indicated proteins in Figure 4A to 4C.

(B) Immunoblotting of indicated proteins in *VHL*-deficient or wildtype HEK293T cells (*VHL*^-/-^ or *VHL*^+/+^).

(C) qPCR analysis of *GLUT1*, *PGK1, PDK1, LDHA, BNIP3, PHD3* and *PKM2* mRNA in *VHL*-deficient or wildtype HEK293T cells (*VHL*^-/-^ or *VHL*^+/+^). Data show mean + SD; Student’s two tailed t-test. *p<0.05, **p<0.01, ***p<0.001, ****p<0.0001. Data from three independent experiments.

(D) Immunoblotting of indicated proteins in Figure 4D to 4F.

(E) Immunoblotting of exogenous Myc-HIF1α expression in HEK293T cells transfected with an increasing amount of HA-SMYD3 expression plasmid (HA empty vector [-] was used as a control).

(F) Immunoblotting of exogenous HA-HIF1α-DM (encoding the double mutant of HIF1α [P402A/P564A]) expression in HEK293T cells transfected with an increasing amount of Flag-SMYD3 expression plasmid (Flag empty vector [-] was used as a control).

**Figure S6. SMYD3 binds to and stabilizes HIF1α independent of its methyltransferase activity.**

(A) Co-immunoprecipitation of HA-SMYD3-F183A with endogenous HIF1α. HEK293T cells were transfected HA-SMYD3-F183A (HA empty vector [-] was used as a control) for 24 h and then cultured hypoxia (1% O_2_) for 4 h. Anti-HA antibody-conjugated agarose beads were used for immunoprecipitation, and the interaction was detected by immunoblotting with the indicated antibodies.

(B) Immunoblotting of endogenous HIF1α expression in H1299 cells transfected with an increasing amount of HA-SMYD3-F183A (HA empty vector [-] was used as a control) and then cultured hypoxia (1% O_2_) for 4 h.

**Video S1**. *Smyd3*^-/-^ zebrafish (right flask) and their wildtype siblings (left flask) stayed in the hypoxia workstation (5% O_2_) for 2 h.

**Video S2**. *Smyd3*^-/-^ zebrafish (right flask) and their wildtype siblings (left flask) stayed in the hypoxia workstation (5% O_2_) for 5 h.

**Supplemental Table S1. The** **quantitative real-time PCR primer sequences and shRNA target sequences**

| Primers | Sequence (5’ to 3’) |
| --- | --- |
| Human-*18SrRNA*-RT-F | GAATTCCCAGTAAGTGGGGG |
| Human-*18SrRNA* -RT-R | GGGCAGGGACTTAATCAACG |
| Human-*PGK1*-RT-F | TGGCTTCTGGCATACCTGCT |
| Human-*PGK1*-RT-R | GCTGCTTTCAGGACCACAGCT |
| Human-*LDHA*-RT-F | AGGAAGAACAGACCCCCCAG |
| Human-*LDHA*-RT-R | CAGCACCAACCCCAACAACT |
| Human-*GLUT1*-RT-F | TGTGGGCCTTTTCGTTAACC |
| Human-*GLUT1*-RT-R | ATCATCAGCATTGAATTCCGC |
| Human-*VEGF*-RT-F | TGTGCCCACTGAGGAGTCC |
| Human-*VEGF*-RT-R | GGTTTGATCCGCATAATCTGC |
| Human-*PDK1*-RT-F | GATGCTAAAGCTATTTATGACT |
| Human-*PDK1*-RT-R | GGAATGACATCATTGTGTCGG |
| Human-*PKM2*-RT-F | GCCTGCTGTGTCGGAGAAG |
| Human-*PKM2*-RT-R | CAGATGCCTTGCGGATGAATG |
| Human-*BNIP3*-RT-F | CTTCCATCTCTGCTGCTCTC |
| Human-*BNIP3*-RT-R | GTAATCCACTAACGAACCAAGTC |
| Human-*PHD3*-RT-F | GAGAGGTCTAAGGCAATGG |
| Human-*PHD3*-RT-R | GGCTCCACATCTGCTATG |
| Human-SMYD1-RT-F | GCCCAATGAGAACATCAGGCT |
| Human-SMYD1-RT-R | GTTGCAGTTAATCACTCCGAAGA |
| Human-SMYD2-RT-F | CTCCAAGCATCTCGGATTCCC |
| Human-SMYD2-RT-R | TGCAACATCAGGAAATATCGCTG |
| Human-SMYD3-RT-F | CCCCACCTCTTACTGCGAG |
| Human-SMYD3-RT-R | TGGCAACGGAAACAGTCACAT |
| Human-SMYD4-RT-F | GGTGGGAAAGGACTCGGAC |
| Human-SMYD4-RT-R | GGTTAGCATGACACAGTGACAT |
| Human-SMYD5-RT-F | GGGAGACCATCTTCGTAGAACG |
| Human-SMYD5-RT-R | ACAGGCTCGGTAGCGATAAAG |
| Human-SETD1A-RT-F | TTGCCATGTCAGGTCCAAAAA |
| Human-SETD1A-RT-R | CGTACTTACGGCACATATCCTTC |
| Human-SETD1B-RT-F | GCTGTCGGTGCCCAAATTC |
| Human-SETD1B-RT-R | CTCAGGAAGTTTTCACGGATGTT |
| Human-SETD2-RT-F | TGCTTCTAGTCGATTTTTGCCC |
| Human-SETD2-RT-R | AGGGTTTGGAGTATCACTTTGC |
| Human-SETD6-RT-F | GGAGAGCTGTTGTTCGTGGT |
| Human-SETD6-RT-R | GAGCGCAAAGTAGGGCCTC |
| Human-SETD8-RT-F | ACAAATGCTCTGGAATGCGTT |
| Human-SETD8-RT-R | CCGGCTAATGGTTTCCCCTG |
| Human-SETDB1-RT-F | AGGAACTTCGGCATTTCATCG |
| Human-SETDB1-RT-R | TGTCCCGGTATTGTAGTCCCA |
| Human-SETDB2-RT-F | GGAGTCTACGAAACGTGGAGG |
| Human-SETDB2-RT-R | CGAGCCAACTGAACATAGGTATT |
| Human-NSD1-RT-F | GAGCTACCTGTCCTTAGGAGAA |
| Human-NSD1-RT-R | GACTCAGGATCATTTGTGCAGT |
| Human-EZH1-RT-F | ATGCGACTTCGACAACTTAAACG |
| Human-EZH1-RT-R | GGCTTCATTGACTGAACAGGTT |
| Human-EZH2-RT-F | AATCAGAGTACATGCGACTGAGA |
| Human-EZH2-RT-R | GCTGTATCCTTCGCTGTTTCC |
| Human-SUV39H2-RT-F | TACTCGTCTTCCCCGAATAGC |
| Human-SUV39H2-RT-R | GGCTGTGGTCAATAGAATCTGAA |
| Human-SUV420H1-RT-F | GAGAAATGGAGGCAAGTTGTCT |
| Human-SUV420H1-RT-R | ACATAGCGACTCTGTCCTTCA |
| Human-SUV420H2-RT-F | TCGGTTTCCGCACCCATAAG |
| Human-SUV420H2-RT-R | CGGAGGTAGCGATAGACGTG |
| Human-PRDM9-RT-F | CAGCCAACAATGGATACTCCTG |
| Human-PRDM9-RT-R | CTGGCCGTATTCATCCCCA |
| Human-METTL22-RT-F | ATGTCCACCTCTATACCCCGA |
| Human-METTL22-RT-R | GGCTCCTTTGTGTGAACATCTC |
| Mouse-*β-actin*-RT-F | TGGAATCCTGTGGCATCCATGAAAC |
| Mouse-*β-actin*-RT-R | TAAAACGCAGCTCAGTAACAGTCCG |
| Mouse-*Glut1*-RT-F | GGAGAGGTGTCACCTACAGC |
| Mouse-*Glut1*-RT-R | AAGGATGCCAACGACGATTC |
| Mouse-*Pgk1*-RT-F | GGAGAGTCCAGAGCGACCCT |
| Mouse-*Pgk1*-RT-R | GCAACTTTAGCGCCTCCCA |
| Mouse-*Vegf*-RT-F | TGACAAGCCAAGGCGGTGAG |
| Mouse-*Vegf*-RT-R | CTCCTCTTCCTTCATGTCAG |
| Zebrafish-*β-actin*-RT-F | TACAATGAGCTCCGTGTTGC |
| Zebrafish-*β-actin* -RT-R | ACATACAATGGCAGGGGTGTT |
| Zebrafish-*pdk1*-RT-F | TGAACCAGCACACTCTTCTG |
| Zebrafish-*pdk1*-RT-R | AGCATCTTTTACCACATCCG |
| Zebrafish-*vegf*-RT-F | TGCTCCTGCAAATTCACACAA |
| Zebrafish-*vegf*-RT-R | ATCTTGGCTTTTCACATCTGCAA |
| Zebrafish-*phd3*-RT-F | CGCTGCGTCACCTGTATT |
| Zebrafish-*phd3*-RT-R | TAGCATACGACGGCTGAACT |
| Zebrafish-*smyd3*-RT-F | GATATCCTCGCTCCCAGTAAAG |
| Zebrafish-*smyd3*-RT-R | TCCAGACAGCATCTCACAATC |
| Human -SMYD3-shRNA#1 | GCGTGTGTCTTTGTTGAAT |
| Human -SMYD3-shRNA#2 | CATCTACCAGCTGAAGGTG |
